# Exploring the Antibacterial Potential of Bis-MPA Dendrimers Bearing Cu(II) and Zn(II) Complexes

**DOI:** 10.1021/acs.biomac.6c00752

**Published:** 2026-07-07

**Authors:** Yoel Garrosa-Miró, Paula Ortega, F. Javier de la Mata, Michael Malkoch, Natalia Sanz del Olmo

**Affiliations:** † Faculty of Sciences, Department of Organic and Inorganic Chemistry, and Research Institute in Chemistry “Andrés M. Del Río” (IQAR), 16720 University of Alcala, 28805 Madrid, Spain; ‡ Networking Research Centre on Bioengineering, Biomaterials and Nanomedicine (CIBER-BBN), 28029 Madrid, Spain; § Institute “Ramón y Cajal” for Health Research (IRYCIS), 28034 Madrid, Spain; ∥ Department of Fibre and Polymer Technology, KTH Royal Institute of Technology, Teknikringen 56-68, 100 44 Stockholm, Sweden

## Abstract

The rise of multidrug-resistant bacteria demands antimicrobial agents that act rapidly and durably. In this work, we aim to develop biodegradable and biocompatible antimicrobial systems by combining the versatile bis-MPA dendritic skeleton with the antibacterial properties of Zn­(II) and Cu­(II) complexes. The resulting metallodendrimers were synthesized using scalable bis-MPA scaffolds and systematically evaluated for antibacterial activity, cytotoxicity, resistance development and bactericidal kinetics. The dendrimers exhibit broad-spectrum antibacterial efficacy at low micromolar concentrations, with minimum inhibitory concentration values as low as 0.4 μM, while maintaining over 70% viability in fibroblasts and keratinocytes and showing no hemolytic activity toward erythrocytes at bactericidal doses. Second-generation Cu­(II) and Zn­(II) bis-MPA dendrimers eradicated *Bacillus subtilis* within 24 h and suppressed *Pseudomonas aeruginosa* within 4 h, without detectable resistance emergence and with superior activity to ciprofloxacin in the latter. Overall, the combination of rapid antimicrobial action, favorable cytotoxicity profiles, strain-dependent resistance behavior, and scaffold versatility highlights bis-MPA metallodendrimers as promising candidates for next-generation antimicrobial therapies.

## Graphical abstract

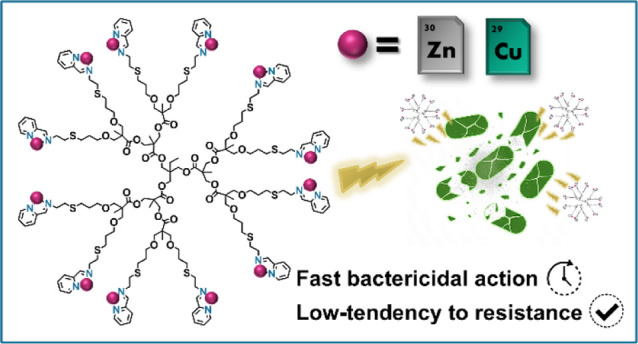

## Introduction

One of the most pressing challenges of the twenty-first century is the emergence of antimicrobial resistance (AMR) against existing treatments, which has resulted in increasing morbidity and mortality worldwide.^1^ It is projected that, by 2050, deaths attributable to AMR could reach 10 million annually.^2^ This alarming trend underscores the urgent need to identify and develop novel antibacterial agents that can replace or complement current therapies to effectively address this global health crisis.

Metal complexes occupy a central position among current antimicrobial agents. While metals have been employed for medicinal purposes since ancient civilizations,^3^ systematic research into their antibacterial properties began in the 19th century, following the discovery of arsenic complexes exhibiting bactericidal activity.^4,5^ This breakthrough paved the way for the identification of complexes containing bismuth, antimony, and, most notably, silver, all exhibiting potent antibacterial effects.^6^ Driven by the need for more accessible, less toxic, and equally potent alternatives, copper and zinc emerged as particularly promising candidates. These metals are not only naturally present in organisms as components of various biomolecules^7^ but can also form complexes with strong antibacterial activity.^8,9^

However, conventional antibiotics and metallodrugs, as well as their administration methods, often face challenges related to hydrophilic–lipophilic balance and require higher doses to overcome bacterial infections, which increase the risk of inducing AMR. Nanotechnology offers a powerful response to this challenge.^10–12^ By engineering nanoscale systems that modulate drug distribution, stability, and local concentration, it becomes possible to enhance the performance of traditional treatments. Prominent examples include ZnO and CuO nanoparticles,^13^ which exploit intrinsic metal-based bioactivity while benefiting from nanoscale dimensions. However, their limited structural precision and surface tunability often restrict precise control over biological interactions at the molecular level.

To address these limitations, dendrimers emerge as highly precise nanoplatforms. Their monodispersity, highly branched architecture, with exact control over size, topology, and multivalent surface groups, enables tailored interactions with bacterial membranes and the environment at the nanoscale.^14^ Their unprecedented structural control, combined with their inherent biocompatibility, renders dendrimers exceptionally versatile nanoplatforms suited for diverse biomedical applications.^15,16^ Their structural features can be precisely engineered to govern specific interactions, such as targeted drug delivery or enhanced antimicrobial activity, providing a robust framework for advanced therapeutic design. One effective strategy to functionalize dendrimers is surface modification with metal complexes, leading to the formation of metallodendrimers, hybrid nanosystems that merge the structural precision of dendrimers with the potent bioactivity of metal-derived motifs, resulting in versatile platforms with wide-ranging biomedical applications.^10^ In the context of metallodendrimers toward bacterial infections, the literature is largely dominated by Cu­(II)- and Zn­(II)-based systems built on well-established dendritic scaffolds. Notable examples include carbosilane frameworks,^17,18^ Poly­(amidoamine) (PAMAM)^19^ and Polypropylenimine (PPI).^20,21^ These systems have been widely evaluated against both Gram-positive and Gram-negative bacteria, with a strong emphasis on biofilm inhibition. Notably, a first generation carbosilane Cu­(II) dendrimer bearing iminopyridine ligands have shown particularly strong anti-*Staphylococcus aureus* biofilm effects.^18^ In parallel, PAMAM- and PPI-based architectures functionalized with naphthalimide or acridine derivatives have demonstrated enhanced antibacterial performance upon Cu­(II) coordination,^19,21^ and in several cases Zn­(II) and Cu­(II) complexes have also enabled photodynamic or light-enhanced antibacterial activity.^22^

Despite the wide range of metallodendrimers reported in the literature with potential in biomedicine, clinical translation remains severely constrained, largely due to high production costs. These expenses arise from the combination of costly metal centers and the challenging, low-yield synthesis required to generate monodisperse dendritic structures. In this context, polyester dendrimers based on 2,2-bis­(hydroxymethyl)­propionic acid (bis-MPA) have been recognized as promising nanoplatforms due to their commercial availability, straightforward and scalable synthesis, and biodegradability.^23,24^ Their degradation yields nontoxic byproducts, which, together with their superior biocompatibility compared to PAMAM dendrimers, a benchmark platform in the biomedical dendrimer field,^25^ render these polyester dendrimers a cost-effective alternative to other dendritic platforms. Several of these bis-MPA dendrimers have been investigated for their antibacterial potential, demonstrating notable activity against pathogens including *Pseudomonas aeruginosa*, *Escherichia coli*, and *Staphylococcus aureus*.^26–28^ However, within the extensive biomedical metallodendrimer literature, examples based on bis-MPA dendritic scaffolds are still relatively scarce. Early examples included Zn-^29^ and Pt-based^30^ systems for catalysis and photonics; however, it was not until around 2010 that these platforms began to be applied in biomedical contexts, particularly in radiolabeling and imaging.^31,32^ In this field, they have also been employed as encapsulating agents for metal complexes based on silver, gadolinium, or dysprosium, enabling applications as antibacterial materials and Magnetic Resonance Imaging (MRI) agents.^33,34^

Taken together, the available literature highlights a gap in the field. Whereas other dendritic scaffolds have been widely engineered for the peripheral coordination of metal complexes to eradicate bacterial infections, bis-MPA dendrimers have been comparatively underexplored for the construction of well-defined metallodendritic systems. In light of these considerations, this work introduces bis-MPA-based polyester metallodendrimers functionalized with Zn­(II) and Cu­(II) complexes in all dendritic branches, leveraging their potent antibacterial activity with low cost, and favorable biocompatibility and biodegradability. These metallodendrimers may serve as candidates for antibacterial applications with potential for large-scale implementation. Their behavior in aqueous solutions at different physiological pH values was investigated, complemented by *in vitro* assays against both Gram-positive and Gram-negative bacteria, and cytotoxicity toward fibroblasts, keratinocytes and erythrocytes, demonstrating their therapeutic potential and safety profile.

## Experimental Section

### Materials

#### Reagents

The following reagents were used as received: triethylamine (≥99%, ThermoFisher, CAS 121-44-8), 2-pyridinecarboxaldehyde (Sigma-Aldrich, CAS 1121-60-4), anhydrous magnesium sulfate (Panreac-Applichem, CAS 7487-88-9), zinc chloride (Fluorochem, CAS 7646-85-7), copper­(II) nitrate trihydrate (Fluorochem, CAS 10031-43-3), and ciprofloxacin (98%, Sigma-Aldrich, CAS 85721-33-1).

#### Solvents

The following solvents were used as received: dichloromethane (DCM) stabilized with ∼50 ppm amylene (Scharlau, CAS 74-09-2), chloroform (CHCl_3_) stabilized with ethanol (Scharlau, CAS 67-66-3), methanol (MeOH, ≥99.8%, Honeywell-Riedel-de Haën, CAS 67-56-1), acetone (Scharlau, CAS 67-64-1), deuterated methanol (CD_3_OD, 99.8%D, Eurisotop, CAS 811-98-3), and deuterated dimethyl sulfoxide (DMSO-*d*_6_, Eurisotop, CAS 2206-27-1).

#### Bacterial Cell Culture

All materials, culture media, and bacterial strains were provided by the Centre for Chemical and Microbiological Analysis, part of the Research Support Centres in Chemistry at the University of Alcalá. Bacteria were cultured in Mueller–Hinton broth (Scharlau, 02-136-500) or on tryptic soy agar (Scharlau, 01-200-500). The bacterial strains, obtained from ATCC (American Type Culture Collection), included *Escherichia coli* ATCC 11775 (*E. coli* 11775); *Pseudomonas aeruginosa* ATCC 9027 (*P. aeruginosa* 9027); *Staphylococcus aureus* ATCC 65388 (*S. aureus* 65388); and *Bacillus subtilis* ATCC 6633 (*B. subtilis* 6633).

#### Cell Cultures

All material, culture media and cell lines were provided by the Cell Culture Unit as part of the Research Support Centre in Medicine-Biology at Alcala University. The nontumoral human foreskin fibroblast cell line HFF-1 (ATCC) and the human epidermal keratinocyte cell line HaCaT (ATCC) were employed in cytotoxicity studies. Cells were cultured in Dulbecco’s Modified Eagle’s Medium (DMEM) supplemented with 4.5 g/L glucose, sodium bicarbonate, l-glutamine, and sodium pyruvate (Sigma-Aldrich, D6429), and 10% sterile-filtered Fetal Bovine Serum (FBS, Sigma-Aldrich, F7524). Cultures were maintained in the presence of antibiotic-antimycotic solution (100×; 10000 U/mL penicillin, 10 mg/mL streptomycin, 25 μg/mL amphotericin B; Sigma-Aldrich, A5955). Dulbecco’s Phosphate-Buffered Saline (PBS, 10×; Sigma-Aldrich, D1408) and Trypsin–EDTA solution (10×; 5 g/L trypsin, 2 g/L EDTA·4Na in 0.9% NaCl; Sigma-Aldrich, T4174) were used for routine cell maintenance. Dimethyl sulfoxide (DMSO) and 3-(4,5-dimethylthiazol-2-yl)-2,5-diphenyltetrazolium bromide (MTT; Sigma-Aldrich, CAS 57360-69-7) were used for cytotoxicity assays.

For hemolysis and hemagglutination assays, defibrinated sheep blood (Thermo Scientific, SR0051C), Dulbecco’s Phosphate-Buffered Saline (PBS, 10×; Sigma-Aldrich, D1408), Triton X-100 (United States Biochemical Corp., 22686) and Concanavalin A from *Canavalia ensiformis* (Sigma-Aldrich, CAS 11028-71-0) were used.

### Synthetic Protocols

The bis-MPA-based dendrimers functionalized with cysteamine (G_*n*_-[Cys]_*m*_) used as starting materials were synthesized according to previously reported protocols.^26^ All synthetic procedures for the dendritic ligands and metallodendrimers are provided in the Supporting Information.

### Characterization Methods

#### NMR Spectroscopy

Mono- and bidimensional NMR experiments (^1^H NMR, ^13^C-{^1^H}-NMR, {^1^H–^1^H}-COSY-2D NMR, {^1^H–^13^C}-HSQC-2D NMR and {^1^H–^13^C}-HMBC-2D NMR) were performed on a Bruker AVANCE NEO 400 MHz spectrometer, with automatic locking and shimming. ^1^H NMR spectra were recorded with 16 scans, 1.0 s of relaxation delay (rd) and a spectral window (sw) of 8196.7 Hz. ^13^C-{^1^H}-NMR spectra were recorded with 1024 scans, 2.0 s of rd and sw of 23809.5 Hz. {^1^H–^1^H}-COSY-2D NMR spectra were recorded with 1 scan, 2.0 s of rd and sw of 5263.2. {^1^H–^13^C}-HSQC-2D NMR and {^1^H–^13^C}-HMBC-2D NMR spectra were recorded with 8 scans, 1.0 s of rd and sw of 4854.4 and 24149.5 Hz, respectively. Samples were analyzed in deuterated solvents: CD_3_OD and DMSO-*d*_6_. ^1^H NMR spectra were referenced to the residual solvent peak of CD_3_OD δ 3.31 ppm and DMSO-*d*_6_ δ 2.25; and ^13^C-{^1^H}-NMR spectra to CD_3_OD of δ 49.00 ppm and DMSO-*d*_6_ δ 39.52. All NMR spectra were processed by MestReNova software.

#### Elemental Analyses

Elemental analyses were carried out using an EA3100 INTEC instrument. Data were processed by Weaver software. The theoretical values for the percentage of each element were calculated using ChemDraw software.

#### UV–Vis Spectroscopy

All experiments were performed on a PerkinElmer UV–vis Lambda 365+ spectrometer. Samples were analyzed as solutions at a concentration of 1.0 mg/mL in Milli-Q water. Electronic spectra were recorded over the wavelength (λ) range of 200–900 nm with a scan rate of 240 nm/min. Absorbance was measured in absorbance units (AU) within the range of 0.00–3.00 AU. Data was analyzed with PerkinElmer UV WinLab software.

### Degradation Evaluation by NMR

Solutions of G_2_-[Zn]_12_ metallodendrimer (25 mg/mL) were prepared in deuterated PBS D_2_O with 1% DMSO-*d*_6_. The pH of the solutions was adjusted to 5.4, 6.4, and 7.4 using NaOD. The solutions were kept at 37 °C throughout the experiment and analyzed by ^1^H NMR at multiple time points (0 h, 1 h, 2 h, 4 h, 6 h, 1 day, 2 days, 7 days, 15 days, and 30 days), using a Varian 500 MHz multinuclear NMR spectrometer.

### Minimum Inhibitory Concentration (MIC) and Minimum Bactericidal Concentration (MBC)

MIC and MBC assays were performed using solutions of metallodendrimers G_*n*_-[Zn]_*m*_ y G_*n*_-[Cu]_*m*_ prepared at concentrations ranging from 1024 to 0.25 ppm. Subsequently, in a 96-well microplate, each well was loaded with 100 μL of one of these solutions, 100 μL of double concentration Mueller–Hinton medium and 5 μL of a bacteria suspension at concentration of 2 × 10^7^ CFU/mL, and microplates were incubated at 37 °C for 20 h. MIC was considered as the minimal concentration at which no turbidity was observed. To determine MBC, 5 μL were taken from each well of the microplate previously used for MIC determination and plated onto fresh TSA plates. The plates were incubated at 37 °C for 20 h. The MBC was considered as the minimal concentration at which no bacterial growth was observed. Negative controls included medium alone, bacterial suspension without biocide, and biocide without bacterial inoculum. All assays were performed in triplicate (*n* = 3).

### Cytotoxicity Evaluation

The cytotoxicity evaluation was carried out toward fibroblasts (HFF-1 cell line) and keratinocytes (HaCaT cell line) using MTT assay. Cells were cultured in tissue culture flask at 37 °C in a humid environment with 5% CO_2_ with Dulbecco’s Modified Eagle Medium (DMEM) supplemented with 10% fetal bovine serum (FBS) and 1% antibiotic/antifungal mixture (penicillin, streptomycin and amphotericin B). Cells were harvested and seeded into 96-well microplate at a concentration of 1 × 10^5^ cell/mL in 100 μL of DMEM and cultured during 24 h to allow adherence. Then, the medium was removed, and cells were treated with 100 μL of metallodendrimers G_*n*_-[Zn]_*m*_ and G_*n*_-[Cu]_*m*_ solutions in DMEM at different concentrations (0.1, 1, 5, 10, and 50 μM). Afterward, cells were cultured with the treatments for 24 h and then incubated with MTT at a final concentration of 0.3 mg/mL for 90 min. Finally, the medium was removed, and the formazan salts were dissolved 100 μL of DMSO. The absorbance of the wells was measured at a wavelength of 595 nm in a microplate reader. Cells cultured in DMEM medium without treatments were used as negative control. Three assays were performed for each sample (*n* = 3). Data were analyzed with GraphPad Prism software.

### Hemolysis Assay

The hemolytic activity of the metallodendrimers was evaluated according to the ISO 10993-4, adapted from previous reports.^18^ Red blood cells (RBCs) were isolated from defibrinated sheep blood by centrifugation at 800*g* for 10 min, followed by four washes with PBS 1×. The resulting RBCs pellet was resuspended in 2 mL of PBS 1×. Subsequently, a 1:50 dilution of RBCs in PBS was prepared and incubated at 37 °C for 15 min under orbital shaker at 100 rpm. Metallodendrimer solutions were prepared in PBS 1× at 1–500 μM. Afterward, 20 μL of each solution were added to 180 μL of incubated RBC suspensions (1:10 dilution), yielding final concentrations of 0.1–50 μM, and the mixtures were incubated at 37 °C for 2 h on an orbital shaker at 100 rpm. After incubation, tubes were centrifuged at 800*g* for 15 min, and 100 μL of the supernatant (H_*x*_) was transferred to a 96-well plate. Free hemoglobin release was quantified by measuring absorbance at 540 nm using a Biotek Epoch 2 microplate reader. The following controls were included in the assay: compound control (H_c_), consisting of 20 μL of each treatment solution added to 180 μL of PBS 1×; negative control (H_0_), consisting of 20 μL of PBS 1× added to 180 μL of incubated RBCs suspension; and positive control (H_70_), consisting of 20 μL of a solution of Triton X-100 in PBS 1× at 1% added to 180 μL of incubated RBCs suspension. Three assays were performed for each sample (*n* = 3). Data were analyzed with GraphPad Prism software. The percentage of hemolysis (% H) was calculated using the following equation, assuming that the positive control induced 70% hemolysis of RBCs

$$\%\;H=\frac{\left(AbsH_{x}-AbsH_{C}\right)}{\left(AbsH_{70}-AbsH_{0}\right)}\times 70$$

### Hemagglutination Assay

The hemagglutination capacity of the metallodendrimers was evaluated according to previously reported protocols.^35^ Supernatants from the hemolysis assay were transferred back into their original Eppendorf tubes. Aliquots of 200 μL were then placed in a round-bottom 96-well plate and incubated at room temperature for 16 h. A negative hemagglutination result was indicated by a well-defined erythrocyte pellet at the bottom of the well, whereas a positive result was evidenced by a diffuse matrix. PBS-treated RBC suspensions (H_0_) were used as the negative control. The positive control consisted of 20 μL of Concanavalin A (50 ppm in PBS) added to 180 μL of RBC suspensions. All assays were performed in triplicate (*n* = 3).

### Induction of Resistance Assay

The minimum inhibitory concentration (MIC) of the treatments was determined over sequential cycles.^36^ The initial MIC (MIC_1_) was established following the protocol described above. For subsequent cycles (MIC_*n*+1_, MIC_*n*+2_...MIC_*n*_), 100 μL from wells treated at a concentration half of the previous cycle MIC were collected and incubated. The bacterial suspension was then adjusted to 2 × 10^7^ CFU/mL, and the MIC was determined again using the same procedure. Results were expressed as the MIC_*n*_/MIC_1_ ratio, defined as the relationship between the MIC value obtained after the final subculture (MIC_*n*_) and that obtained in the initial culture (MIC_1_). Three assays were performed for each sample (*n* = 3).

### Time-Kill Curves Assay

A bacterial suspension (5 × 10^5^ CFU/mL, 2.187 mL) was incubated in 15 mL Falcon tubes at 37 °C on an orbital shaker at 150 rpm until the exponential growth phase was reached. Each culture was adjusted to a final volume of 2.5 mL by adding 313 μL of an 8 × MIC treatment solution prepared in sterile distilled water. Ciprofloxacin and sterile water were included as positive and negative controls, respectively. Cultures were further incubated under the same conditions, and 200 μL samples were collected at 0, 0.5, 1, 2, 4, 6, and 24 h. Each sample was serially diluted up to 1 × 10^–7^ CFU/mL in 0.9% NaCl saline solution. Aliquots of 5.0 μL from each dilution were plated onto TSA plates, and incubated for 20 h, after which CFU were counted at each time point. The concentration of CFU/mL was obtained following equation

$$\frac{CFU}{mL}=\frac{1}{10^{-D}}\cdot \frac{1}{10^{-2}}$$

where *D* denotes the first dilution at which bacterial growth was observed. Three assays were performed for each sample (*n* = 3).

### Statistical Analysis

Statistical analysis was performed using one-way ANOVA followed by Bonferroni post hoc test. Each treatment group was compared to its corresponding control group. Statistical significance was set at *p < 0.01, **p < 0.001 and ***p < 0.0001. Analyses were performed using GraphPad Prism software.

## Results and Discussion

### Synthesis and Structural Characterization of Zn­(II) and Cu­(II) Polyester Based Metallodendrimers

The development of scalable metallodendritic polyester platforms with antibacterial activity demands robust dendritic scaffolds capable of supporting efficient metal coordination. Guided by these criteria, previously reported bis-MPA–derived cationic dendrimers functionalized with cysteamine groups^26^ were selected as the structural backbone, owing to its enhanced hydrolytic stability in aqueous environments and its suitability for further functional diversification. To impart chelating capacity, iminopyridine Schiff base (SB) ligands were incorporated. These ligands have been introduced in a wide variety of dendritic scaffolds and are known for generating well-defined coordination pockets and for the intrinsic reactivity of the imine bond (−N=CH−), which can engage in hydrogen-bonding interactions with biomolecular targets,^37,38^ thus offering an avenue to modulate and potentially amplify antibacterial performance. As metal precursors salts, ZnCl_2_ and Cu­(NO_3_)_2_ were deliberately selected, based on compelling evidence demonstrating an optimal balance between solubility in dendritic complexes and proven antibacterial activity.^17,18^ These features position them as suitable candidates for generating the Zn­(II)- and Cu­(II)-based platforms explored in this work.

With this rationale in place, bis-MPA-based Cu­(II) and Zn­(II) metallodendrimers were synthesized from the first to the third dendritic generation, incorporating between 6 and 24 peripheral metal complexes. The overall synthetic strategy is summarized in Figure 1A. For clarity and consistency throughout the text, the following nomenclature is adopted: G_*n*_-[Lig/M]_*m*_, where G denotes the dendritic generation built from a trimethylolpropane (TMP) core, such that for *n* = 1, *m* = 6; *n* = 2, *m* = 12; and *n* = 3, *m* = 24. The square brackets indicate either the ligand (Lig)—cysteamine (Cys), neutral amine (NH_2_), or iminopyridine (NPh)—; or the metal complex (M) with Zn­(II) or Cu­(II) centers (Figure 1B).

**Figure 1:**
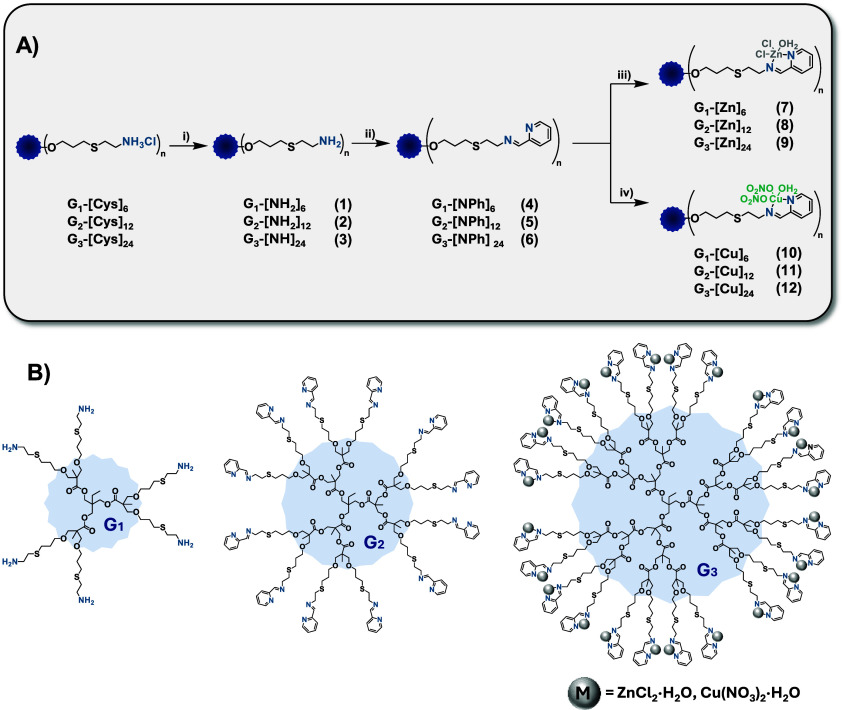
(A) Synthetic route to bis-MPA Zn­(II) and Cu­(II) metallodendrimers: (i) Et_3_N, DCM/H_2_O, rt, 2 h (ii) 2-pyridincarboxaldehyde, DCM, rt, 30 min (iii) ZnCl_2_, MeOH, rt, overnight. (iv) Cu­(NO_3_)_2_, acetone, rt, overnight. (B) Representative structures of dendrimers up to third generation: G_1_-[NH_2_]_6_ (**1**), G_2_-[NPh]_12_ (**5**) and G_3_-[M]_24_, (M = Zn­(II) (**9**) or Cu (II) (**11**)).

The envisioned metallodendritic platforms were synthesized via a sequential approach, allowing for systematic incorporation of chelating ligands and metal centers. Initially, the ammonium groups of the previously reported cysteamine-functionalized dendrimers were neutralized with triethylamine (Et_3_N) in a 1:1 H_2_O/DCM mixture. The resulting neutral derivatives were extracted into the organic phase. The reaction progress was monitored by ^1^H NMR in CD_3_OD, showing a shift of the −C ***H***_**2**_NH_3_^+^ signal from 3.15 to 2.81 ppm, consistent with the neutralization of the ammonium groups. Afterward, SBs functionalities were introduced via condensation with 2-pyridinecarboxaldehyde in DCM in the presence of dry MgSO_4_, yielding imine linkages within 15 min. Reaction completion was confirmed by ^1^H NMR in CD_3_OD through the appearance of a singlet at 8.36 ppm attributed to the imino proton (−N=C ***H***−) and a downfield shift of the −C ***H***_**2**_N=CH– signal to 3.86 ppm, along with aromatic resonances. Similarly, these shifts were also observed in ^13^C NMR spectrum, with the appearance of the imino carbon (−N= ***C*** H−) at 162.5 ppm and the displacement of the −***C*** H_2_N=CH signal from 41.3 ppm in the neutral amine to 60.3 ppm (see Supporting Information, Figures S1–S6).

The resulting SB dendrimers were then employed as ligands for the coordination of ZnCl_2_ and Cu­(NO_3_)_2_ metal salts, yielding both families of polyester-based metallodendrimers through similarly straightforward protocols. Concerning the Zn­(II) metallodendrimers, the addition of the metal salt to the dendritic system in MeOH, led to the formation of a reddish-brown precipitate after 16 h of reaction. Coordination was confirmed by ^1^H NMR in deuterated DMSO, as evidenced by downfield shifts of the aromatic protons as well as the protons near the coordinating nitrogen atoms: −CH_2_N=C ***H***– (from 8.36 to 8.61 ppm) and −C ***H***_**2**_N=CH– (from 3.86 to 3.75 ppm) (Figure 2A). Interestingly, no significant shifts were observed for methylene protons near oxygen or sulfur atoms, indicating that metal coordination occurred exclusively through nitrogen donor sites. Similar results were observed by ^13^C NMR, by downfield shifts of the carbons near the coordinating nitrogen atoms: −CH_2_N= ***C*** H– (from 164.7 to 162.2 ppm) and −***C*** H_2_N=CH– (from 61.4 to 57.0 ppm) (see Supporting Information, Figures S7–S9).

**Figure 2:**
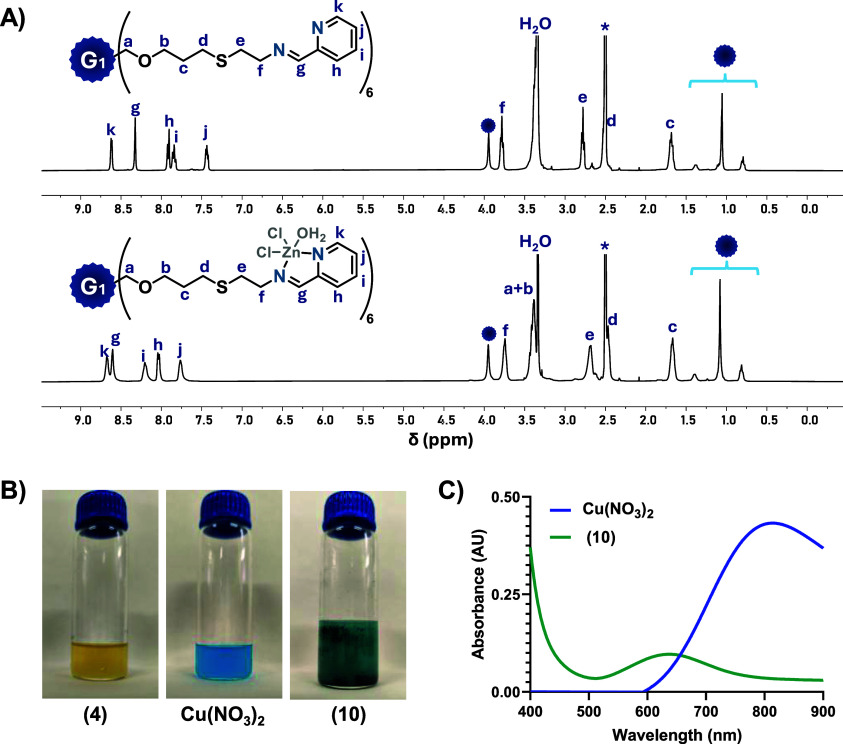
(A) ^1^H NMR 400 Hz spectra of G_1_-[NPh]_6_ (**4**, top) and G_1_-[Zn]_6_ (**7**, bottom) in deuterated DMSO-*d*_6_ (*). (B) Color changes during Cu­(II) coordination in acetone. From left to right: G_1_-[NPh]_6_ (**4**, yellow solution), Cu­(NO_3_)_2_ (blue solution), and G_1_-[Cu]_6_ (**10**, dark green solution with precipitate). (C) UV–vis spectra in water of G_1_-[Cu]_6_ (**10**) and Cu­(NO_3_)_2_.

On the other hand, for the Cu­(II) metallodendrimers, upon mixing the yellow solution of the dendritic ligand G_1_-[NPh]_6_ (**4**) with the blue Cu­(NO_3_)_2_ solution in acetone, resulted in an immediate color change and the formation of a less soluble species, consistent with the generation of the Cu­(II) metallodendrimer G_1_-[Cu]_6_ (**10**) (Figure 2B). Although direct structural characterization by NMR was precluded due to the paramagnetic nature of Cu­(II), the NMR spectra showed significant line broadening of the dendritic backbone signals and complete disappearance of the aromatic resonances associated with the peripheral coordinating groups, consistent with coordination at the dendritic periphery (Figure S10). These spectral changes, together with the observed color change and reduced solubility, support metal–ligand complex formation. Additional evidence was obtained by UV–vis spectroscopy, which showed a d–d transition band at 630 nm and the disappearance of the free metal complex absorption band at 810 nm (Figures 2C, and S11).

Finally, elemental analysis of C, H, and N was performed for all metallodendrimers obtained to assess their purity prior to *in vitro* studies. The experimental results matched the expected values, confirming the proposed structures and supporting the use of these compounds in subsequent experiments (see Supporting Information).

### Chemical Behavior in Aqueous and Physiological Media

Following the synthesis of the Cu­(II) and Zn­(II) metallodendrimers, evaluating their behavior in aqueous solutions became a critical first step before exploring their therapeutic potential. Due to the diamagnetic character of the Zn­(II) derivative, the second generation metallodendrimer G_2_-[Zn]_12_ (**8**) was selected as model for the hydrolytical stability assessment by NMR. These studies were carried out at 37 °C, over the course of a month and at pH values of 5.4, 6.4, and 7.4, to closely mimic biological conditions. Although previous reports have demonstrated that these cysteamine-functionalized polyester scaffolds exhibit remarkable hydrolytic stability under such conditions,^26^ the present study specifically determines how the local environment affects the coordination sphere of the metal centers.

The behavior of G_2_-[Zn]_12_ (**8**) was investigated by ^1^H NMR in deuterated PBS solutions, with pH adjusted using NaOD and 1% DMSO-*d*_6_ added to enhance solubility. The system proved insoluble at pH 7.4 at the concentration required for NMR analysis, likely due to the substitution of coordinated H_2_O molecules by hydroxide ligands from the basic medium, leading to precipitation. Consequently, stability assessments focused on pH 6.4 (see Supporting Information, Figure S12) and 5.4 (Figure 3A). Exposure to these acidic media triggered gradual degradation, more pronounced at pH 5.4. This behavior might arise from protonation of the imine nitrogen under acidic conditions, increasing the electrophilicity of the imine carbon and rendering it more susceptible to nucleophilic attack by deuterated water. Consequently, the cleavage of the SB occurs, yielding 2-pyridinecarboxaldehyde, the Zn­(II) complex, and the dendrimer bearing free protonated primary amines. This degradation mechanism is supported by the appearance of the C ***H***_**2**_ND_3_^+^ signal at 3.1 ppm and the attenuation of the C ***H***_**2**_N=CPh signal at 3.75 ppm. Integration of these resonances allows quantification of the SBs remaining over time (Figure 3B). At time zero, the SB content was 78.4% at pH 6.4 and 72.4% at pH 5.4, remaining essentially stable during the first 24 h. Beyond 48 h, however, the SBs proportion gradually decreased in both media, reaching 22% after 30 days. Overall, these results show that Zn­(II) metallodendrimers retain sufficient short-term integrity under mildly acidic conditions to warrant further evaluation as antibacterial agents.

**Figure 3:**
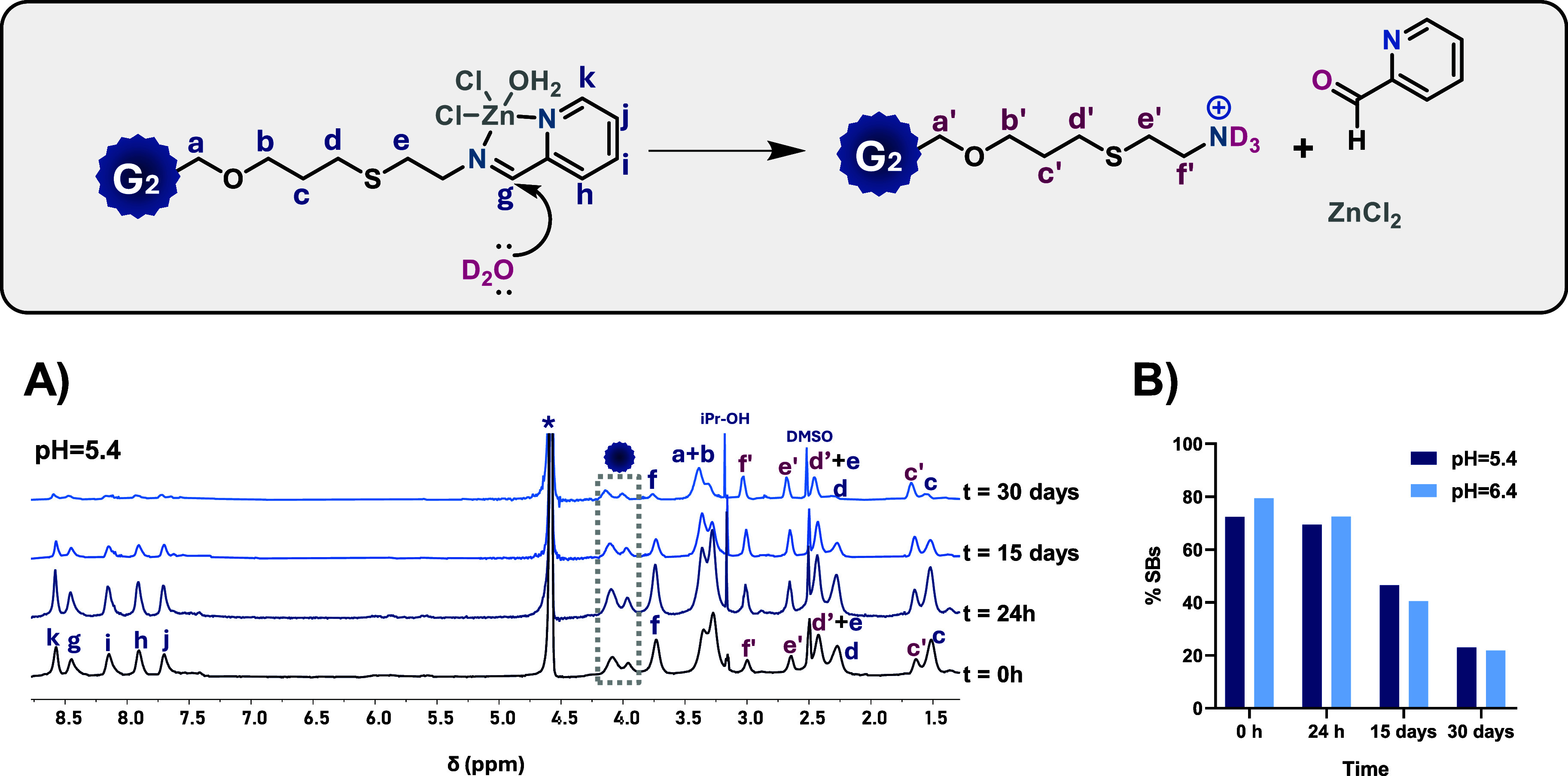
(A) Degradation evaluation of G_2_-[Zn]_12_ (**8**) at pH 5.4 by ^1^H NMR at different time points (0 h, 24 h, 15 days and 30 days). (B) Remaining Schiff bases (SBs) in the dendritic structure of G_2_-[Zn]_12_ (**7**) evaluated in PBS at pH 5.4 and 6.4, measured by ^1^H NMR, expressed as the ratio of the integrals of signals c′/c.

### Antibacterial Activity toward Planktonic Bacteria: MIC and MBC Determination

To assess the antibacterial efficacy of the synthesized dendrimers, MIC and MBC were determined against representative planktonic bacterial strains: *E. coli* and *P. aeruginosa* (Gram-negative), and *S. aureus* and *B. subtilis* (Gram-positive) (Table 1). Overall, most metallodendrimers exhibited both bactericidal and bacteriostatic activity, with the most pronounced effects observed against *P. aeruginosa* and *B. subtilis*. In sharp contrast, *S. aureus* consistently showed the lowest susceptibility, with the highest-generation G_3_ metallodendrimers exceeding 512 ppm of MIC and MBC, indicating a marked reduction in activity despite the expected higher sensitivity of Gram-positive strains. This trend, previously reported for polyester dendrimers,^26,27^ contrasts with the behavior of other dendritic scaffolds, such as carbosilane^17,18,35^ or PAMAM dendrimers,^19^ which typically exhibit their highest activity against Gram-positive bacteria. Remarkably, among Gram-negative bacteria, *P. aeruginosa* displayed a 16-fold higher susceptibility than *E. coli* for compound **8**, highlighting a strong and strain-specific antibacterial profile of the dendritic systems.

**Table 1:** MIC and BIC Values of the Metallodendrimers G_*n*_-[Zn]_*m*_ (*n* = 1, *m* = 6, (**7**); *n* = 2, *m* = 12, (**8**); *n* = 3, *m* = 24, (**9**)) and G_*n*_-[Cu]_*m*_ (*n* = 1, *m* = 6, (**10**); *n* = 2, *m* = 12, (**11**); *n* = 3, *m* = 24, (**12**))

	*E. coli* (Gram (−))					*P. aeruginosa* (Gram (−))				
Compound	MIC		MBC		MBC/MIC	MIC		MBC		MBC/MIC
	[μg/mL]	[μM]	[μg/mL]	[μM]		[μg/mL]	[μM]	[μg/mL]	[μM]	
(**7**)	64	24.3	64	24.3	1	16	6.1	32	12.2	2
(**8**)	128	23.4	256	46.8	2	8	1.4	16	2.8	2
(**9**)	32	2.9	64	5.8	2	64	5.7	64	5.7	1
(**10**)	32	10.8	32	10.8	1	32	10.8	32	10.8	1
(**11**)	16	2.6	16	2.6	1	16	2.6	16	2.6	1
(**12**)	32	2.6	32	2.6	1	32	2.6	64	5.2	2

	*S. aureus* (Gram (+))					*B. subtilis* (Gram (+))				
Compound	MIC		MBC		MBC/MIC	MIC		MBC		MBC/MIC
	[μg/mL]	[μM]	[μg/mL]	[μM]		[μg/mL]	[μM]	[μg/mL]	[μM]	
(**7**)	64	24.3	64	24.3	1	4	1.5	4	1.5	1
(**8**)	128	23.4	256	46.8	2	2	0.4	2	0.4	1
(**9**)	>512	>45.9	>512	>45.9	-	8	0.7	8	0.7	1
(**10**)	256	86.8	256	86.8	1	4	1.4	4	1.4	1
(**11**)	256	41.9	256	41.9	1	4	0.6	4	0.6	1
(**12**)	>512	>41.2	>512	>41.2	-	8	0.6	8	0.6	1

To assess whether the observed antibacterial activity originates from the metal salts or from the metallodendritic architectures, the corresponding ZnCl_2_ and Cu­(NO_3_)_2_ salts were evaluated against the four bacterial strains, yielding MIC and MBC values exceeding 512 ppm in all cases. These results indicate that the antibacterial activity is primarily associated with the metallodendritic systems rather than the metal salts alone. In contrast, the iminopyridine-based dendritic precursors (**4**–**6**) could not be used as controls due to their poor water solubility and hydrolytic instability in aqueous media, resulting from the presence of labile C=N imine bonds that undergo rapid degradation.

Among all metallodendrimers, the second-generation derivatives G_2_-[Zn]_12_ (**8**) and G_2_-[Cu]_12_ (**11**) exhibited the most potent antibacterial activity, with MBC values of 16 ppm against *P. aeruginosa* and 2 and 4 ppm, respectively, against *B. subtilis*. Such behavior is in line with the broader antibacterial dendrimer literature, where activity commonly shows a nonlinear dependence on generation and intermediate architectures often outperform both smaller and larger analogues.^18,27^ In this context, the enhanced activity of the second-generation compounds likely reflects an optimal compromise between multivalency and effective surface presentation of the peripheral Cu­(II) and Zn­(II) complexes, while further growth to G_3_ does not improve activity and may instead reduce efficiency through increased steric crowding and lower accessibility of the active surface. Additionally, no significant differences were observed between the two metal centers, except against *E. coli*, where Cu­(II) metallodendrimers **10–12** exhibited the highest activity. This observation is also consistent with earlier studies showing that the biological behavior of Cu­(II) and Zn­(II) metallodendrimers is strongly scaffold-dependent and cannot be reduced to a simple metal-based ranking.^19,21^

Based on these results, the potential for the tested planktonic bacteria to develop tolerance to the evaluated treatments can be estimated. Tolerance is defined as the ability of bacteria to evade the lethal action of an antimicrobial agent and can be assessed using the MBC/MIC ratio.^39^ Strains are generally considered tolerant when the MBC/MIC ratio is ≥16.^40,41^ In this study, all treatments showed MBC/MIC ratios ≤2, suggesting that none of the bacterial strains tested displayed tolerance.

### In Vitro Cytotoxicity Evaluation

To determine the potential of these metallodendrimers in the biomedical field it is essential to determine their toxicity in noncancer cell lines. Following that rationale, the cytotoxicity of all metallodendrimers was evaluated against the HFF-1 fibroblast cell line using an MTT assay (Figure 4A). A general trend was observed with cytotoxicity increasing alongside dendrimer generation, while no differences were noted between Zn­(II) and Cu­(II) metal centers. These cell viability percentages were compared to the MBC values for each compound. Among them, the second-generation metallodendrimers G_2_-[Zn]_12_ (**8**) and G_2_-[Cu]_12_ (**11**) demonstrated bactericidal activity at concentrations where cell viability remained above 70%,^42^ against *P. aeruginosa* and *B. subtilis*. Based on their favorable balance between antibacterial activity and fibroblast compatibility, G_2_ dendrimers were selected for further evaluation. Cytotoxicity was evaluated against the HaCaT keratinocyte cell line (Figure 4B), where both metallodendrimers were nontoxic at the bactericidal concentrations, in accordance with the results observed in fibroblasts. Finally, hemolysis and hemagglutination assays were performed to investigate the cytotoxic effects of the selected metallodendrimers **8** and **11** on RBCs obtained from defibrinated sheep blood. The results showed that neither compound induced hemolysis at the concentrations tested, with no significant differences relative to the negative PBS control (Figure 4C). In addition, hemagglutinating activity was only observed at a concentration of 50 μM, which is substantially higher than the MBC values determined for both metallodendrimers (Figure 4D). Taken together, these cell viability results highlight the favorable biocompatibility profile of these derivatives, supporting their potential for further biomedical applications.

**Figure 4:**
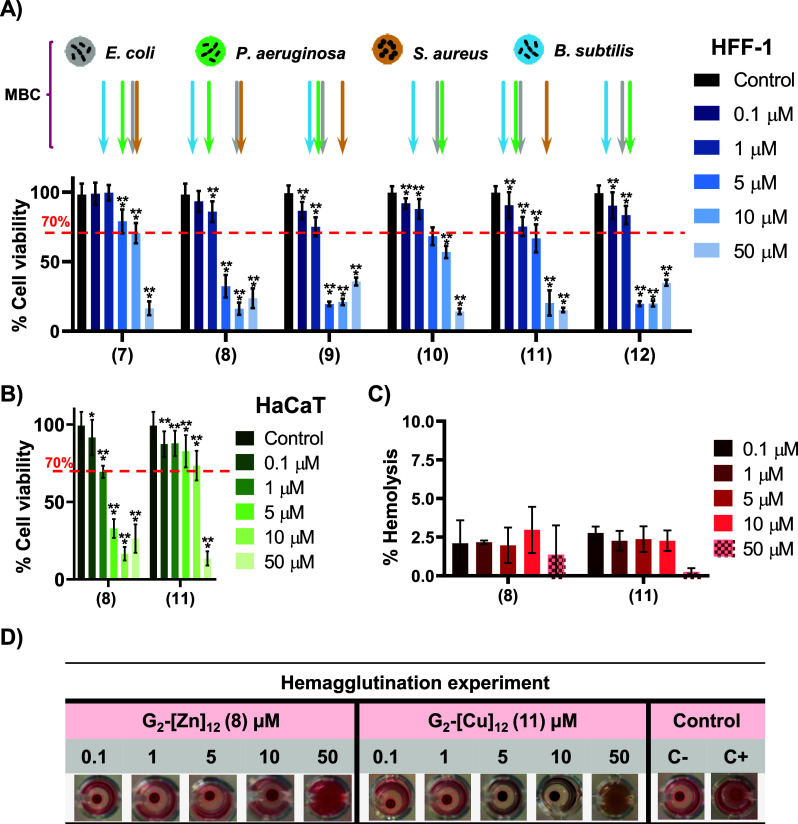
(A) MTT Cytotoxicity assay of compounds G_*n*_-[Zn]_*m*_ (*n* = 1, m = 6, (**7**); *n* = 2, *m* = 12, (**8**); *n* = 3, *m* = 24, (**9**)) and G_*n*_-[Cu]_*m*_ (*n* = 1, *m* = 6, (**10**); *n* = 2, *m* = 12, (**11**); *n* = 3, *m* = 24, (**12**)) against HFF-1 fibroblast cell line. (B) MTT Cytotoxicity assay of compounds G_2_-[Zn]_12_ (**8**) and G_2_-[Cu]_12_ (**11**) against HaCaT keratinocyte cell line. (C) Hemolysis percentages of G_2_-[Zn]_12_ (**8**) and G_2_-[Cu]_12_ (**11**) at the indicated concentrations. PBS 1× served as the negative control and 1% Triton X-100 in PBS served as the positive control. (D) Hemagglutination assay of G_2_-[Zn]_12_ (**8**) and G_2_-[Cu]_12_ (**11**) performed across the indicated concentrations. PBS 1× served as the negative control and Concanavalin A served as the positive control. Mean values are shown with error bars showing standard deviation, *n* = 3. Statistical analysis: One-way ANOVA (Bonferroni). *p < 0.01 vs control, **p < 0.001 vs control and ***p < 0.0001 vs control.

### Induction of Antibiotic Resistance

Building on the global challenge posed by AMR, the ability of the selected dendrimers G_2_-[Zn]_12_ (**8**) and G_2_-[Cu]_12_ (**11**) to prevent the development of bacterial resistance was investigated in *P. aeruginosa* and *B. subtilis*. These strains were chosen as representative Gram-negative and Gram-positive model organisms due to their higher susceptibility to the dendritic compounds observed in the initial MIC/MBC antibacterial screening. This assay involved evaluating the MIC of the two selected metallodendrimers over consecutive cycles against both bacterial strains, using subcultures derived from the MIC of the previous cycle. The number of cycles varied depending on bacterial growth dynamics, with *P. aeruginosa* requiring 10 cycles while *B. subtilis* was limited to 2 cycles due to rapid saturation. Resistance development was assessed by the ratio of the MIC in the final cycle (MIC_n_) to that of the initial cycle (MIC_1_).^36^ In both cases, the broad-spectrum commercial antibiotic ciprofloxacin was used as a control.^43,44^

The results indicated that the behavior of the metallodendrimers depends on the bacterial species tested (Figure 5). Interestingly, both metallodendrimers exhibited high efficacy against the Gram-negative bacterial model *P. aeruginosa*, with no differences observed between MIC_10_ and MIC_1_. In contrast, ciprofloxacin showed a 32-fold increase in MIC relative to the first cycle, rising from 0.25 μg/mL to 8 μg/mL. While MIC_10_ for the antibiotic indicates bacteriostatic activity, it also reveals a clear tendency of *P. aeruginosa* to develop resistance to ciprofloxacin, in sharp contrast to the behavior observed for both second generation metallodendrimers. This behavior is consistent with the broader antibacterial dendrimer literature, in which some multivalent membrane-active dendritic systems show a low propensity to select resistance upon repeated exposure.^45,46^ Conversely, against the Gram-positive bacterial model *B. subtilis*, these metallodendrimers exhibited a completely opposite behavior. A marked increase in MIC values was observed after only two cycles, exceeding the highest concentration tested in the assay (256 μg/mL). This species-dependent outcome is in line with previous reports on other polyester–based dendrimers against Gram-positive bacterial models.^27^ Taken together, these findings highlight the strong dependence of metallodendrimer activity and resistance development on bacterial cell envelope architecture, underscoring their potential as next-generation antimicrobial scaffolds.

**Figure 5:**
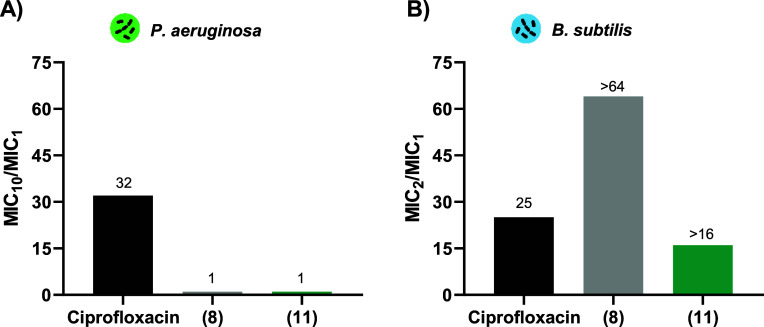
Induction of antibiotic resistance assay of G_2_-[Zn]_12_ (**8**) and G_2_-[Cu]_12_ (**11**). (A) *P. aeruginosa* resistance development expressed as the ratio of the MIC in the 10th subculture to that of the first subculture (MIC_10_/MIC_1_). (B) *B. subtilis* resistance development expressed as the ratio of the MIC in the second subculture to that of the first subculture (MIC_2_/MIC_1_).

### Time-Kill Kinetics of Second Generation Metallodendrimers

Having established both the antibacterial activity and favorable cytotoxicity profile of the synthesized metallodendrimers, we next sought to investigate their bactericidal kinetics against actively growing bacteria using a time-kill assay. Unlike static MIC and MBC determinations, which measure bacterial inhibition primarily during the lag phase, this approach allows the assessment of bacterial population dynamics in the exponential growth phase, more closely mimicking an active infection. Based on the previously described results, second-generation G_2_-[Zn]_12_ (**8**) and G_2_-[Cu]_12_ (**11**) dendrimers were selected as potential antimicrobial candidates against *P. aeruginosa* and *B. subtilis* with ciprofloxacin, serving as a positive control. For this study, the compounds were used at their respective MIC values to assess their antibacterial effects at concentrations previously shown to be nontoxic to fibroblasts.

Both second-generation metallodendrimers exhibited rapid and potent antibacterial activity against *P. aeruginosa* and *B. subtilis*, achieving over 99% reduction of the initial bacterial load within 30 min of administration (Figure 6). Notably, G_2_-[Cu]_12_ (**11**) was more effective against *P. aeruginosa*, whereas G_2_-[Zn]_12_ (**8**) showed superior activity against *B. subtilis*. In *P. aeruginosa*, both compounds exerted a bacteriostatic effect during the first 4 h, followed by bacterial regrowth reaching >10^10^ CFU/mL after 24 h, comparable to the untreated control (Figure 6A). This regrowth is consistent with the expected behavior of single-dose treatments, which often require repeated administration to maintain bactericidal effects, and has been described for other membrane-active dendrimers against Gram-negative bacteria.^47^ In contrast, complete eradication of *B. subtilis* was observed within 24 h, with transient differences between the dendrimers: G_2_-[Zn]_12_ (**8**) displayed a brief increase to 1 × 10^3^ CFU/mL, which was eliminated after 4 h, while G_2_-[Cu]_12_ (**11**) peaked at 1 × 10^6^ CFU/mL at 4 h before full eradication (Figure 6B). This species-dependent kinetic behavior further supports that the antibacterial performance of dendritic systems depends not only on overall potency, but also on how each bacterial envelope responds over time to multivalent membrane-active agents.^48^

**Figure 6:**
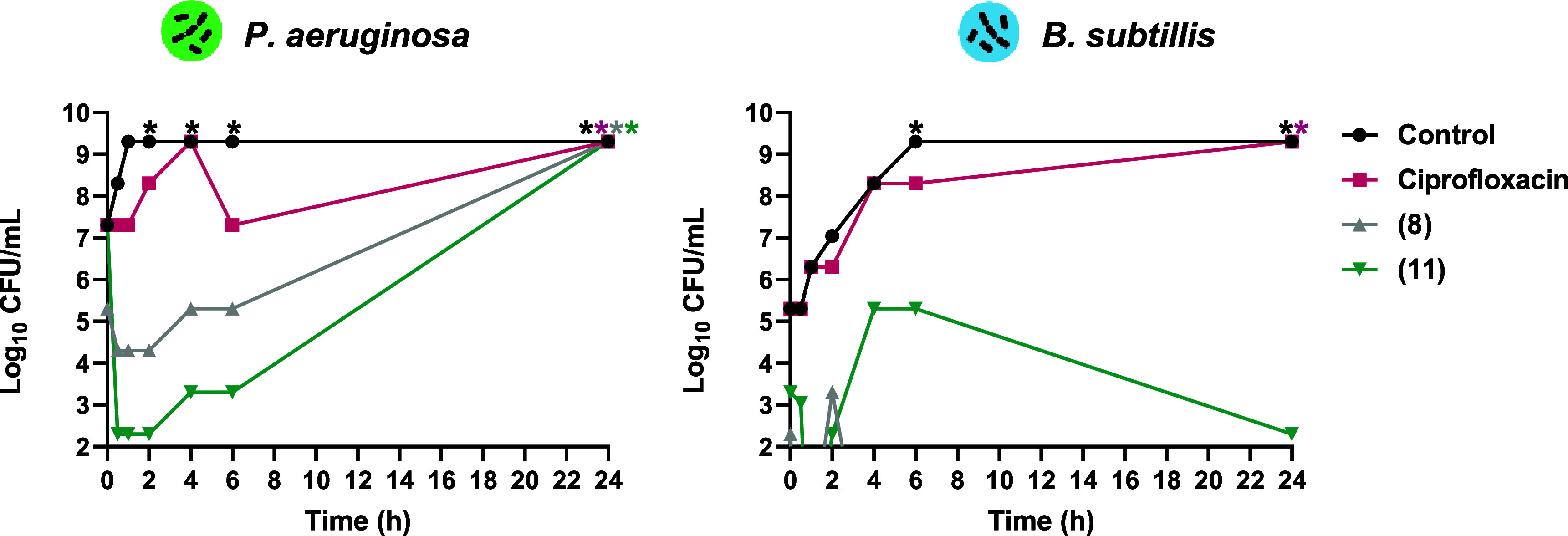
Time-kill assay for compounds G_2_-[Zn]_12_ (**8**) and G_2_-[Cu]_12_ (**11**) in each MIC concentration against *P. aeruginosa* (A) and *B. subtilis* (B). Water was added as negative control and ciprofloxacin was added as positive control in its MIC against each bacteria strain. (*) denotes measurements above the instrument’s upper limit; the real values may exceed those shown in the graphs.

Importantly, both metallodendrimers outperformed ciprofloxacin, which produced only a modest reduction in bacterial population and lacked bactericidal activity under the same conditions. This behavior is consistent with previous reports,^49,50^ where ciprofloxacin requires the administration of a higher antibiotic concentration relative to its MIC in order to achieve full therapeutic effect.^43^

## Conclusions

The unique properties of bis-MPA dendrimers have been successfully integrated with metal complexes, particularly Zn­(II) and Cu­(II), yielding metallodendrimers with remarkable antibacterial potential. Hydrolytic stability studies of the diamagnetic G_2_-[Zn]_12_ at physiologically relevant pH values revealed that the imine bond remained largely intact for up to 24 h, followed by gradual degradation, with 22% of the imines still preserved after 30 days, highlighting their exceptional short-term stability for their use in biomedicine. These metallodendrimers exhibited potent and broad-spectrum antibacterial activity, with highly favorable MIC and MBC values against planktonic bacteria in the micromolar range. Notably, *P. aeruginosa* and *B. subtilis* were especially susceptible, with the G_2_ derivatives (G_2_-[Zn]_12_ and G_2_-[Cu]_12_) emerging as standout candidates, combining maximal efficacy and nontoxicity toward fibroblasts, keratinocytes at RBCs at active bactericidal concentrations. Interestingly, the combined analysis of bacterial resistance and kinetic studies for the two selected metallodendrimers in the most promising strains demonstrates that their antibacterial potential surpasses that of broad-spectrum commercial antibiotics such as ciprofloxacin. Against *P. aeruginosa*, repeated exposure to ciprofloxacin over 10 cycles resulted in a 32-fold increase in MIC, whereas MIC values for the metallodendrimers remained stable, indicating effective inhibition of resistance development. Kinetic studies further showed that these dendrimers produced a substantial suppression of *P. aeruginosa* within just 4 h. Although the bacterial population eventually regrew, repeated dosing could maintain suppression without inducing resistance. In contrast, against *B. subtilis*, the metallodendrimers did allow the development of bacterial resistance, similar to ciprofloxacin. However, kinetic analyses revealed that they could achieve complete eradication of all bacteria, including persistent subpopulations, within 24 h. More broadly, current literature on antibacterial dendrimers remains largely focused on nonmetalated systems, while mechanistic studies such as resistance selection and time-kill kinetics remain scarce for metallodendrimer platforms. The present study highlights the importance of incorporating such dynamic evaluations, as they provide critical insight into bacterial regrowth, resistance emergence, and species-dependent responses that are not captured by MIC and MBC values alone. In this context, the second-generation metallodendrimers reported herein represent a promising platform for the development of metal-containing dendritic systems in antimicrobial research.

## Supplementary Material



## Notes

Synthetic protocols for all dendrimers; ^1^H, ^13^C, and {^1^H–^13^C} NMR spectra; UV–vis spectra of Cu­(II) metallodendrimers; and degradation study of G_2_-[Zn]_12_ monitored by ^1^H NMR (PDF)
