# Supplementary material for: Exploring the Antibacterial Potential of Bis-MPA Dendrimers Bearing Cu(II) and Zn(II) Complexes
Source: Biomacromolecules. 2026 Jul 7;27(8):5461–72. doi: 10.1021/acs.biomac.6c00752 (PMC13463553; doi:10.1021/acs.biomac.6c00752)
Supplement: Supplementary file 1 [file bm6c00752_si_001.pdf]

Electronic Supplementary Information

Exploring the Antibacterial Potential of Bis-MPA Dendrimers Bearing Cu(II) and Zn(II) Complexes

Yoel Garrosa-Miró[1,2,3], Paula Ortega[1,2,3], F. Javier de la Mata[1,2,3], Michael Malkoch[4] and Natalia Sanz del Olmo[1,3,4]*#.*

[1]University of Alcala, Faculty of Sciences, Department of Organic and Inorganic Chemistry, and Research Institute in Chemistry "Andrés M. Del Río" (IQAR), 28805 Madrid, Spain.

[2]Networking Research Center on Bioengineering, Biomaterials and Nanomedicine (CIBER-BBN), Spain.

[3]Institute "Ramón y Cajal" for Health Research (IRYCIS), Spain.

[4]Department of Fiber and Polymer Technology, KTH Royal Institute of Technology, Teknikringen 56-68, 100 44, Stockholm, Sweden.

*** Corresponding authors:** Dr. Natalia Sanz del Olmo and Prof. Michael Malkoch

# Address correspondence to:

Dr. Natalia Sanz del Olmo

Department of Organic Chemistry and Inorganic Chemistry, University of Alcalá. Road Madrid-Barcelona, Km 33, 100, 28805. Alcalá de Henares, Madrid, Spain

Email: natalia.sanzo@uah.es

Table of Contents

Synthesis protocols 3

Figures 10

Figure S1. NMR spectra of G_1-$[NH_2]_6$ **(1)** in CD_3OD 10

Figure S2. NMR spectra of G_2-$[NH_2]_{12}$ **(2)** in CD_3OD..11

Figure S3. NMR spectra of G_3-$[NH_2]_{24}$ **(3)** in CD_3OD.. 12

Figure S4. NMR spectra of G_1-$[NPh]_6$ **(4)** in DMSO-d^6... 13

Figure S5. NMR spectra of G_2-$[NPh]_{12}$ **(5)** in DMSO-d^6 14

Figure S6. NMR spectra of G_3-$[NPh]_{24}$ **(6)** in DMSO-d^6 15

Figure S7. NMR spectra of G_1-$[Zn]_6$ **(7)** in DMSO-d^6 16

Figure S8. NMR spectra of G_2-$[Zn]_{12}$ **(8)** in DMSO-d^6 17

Figure S9. NMR spectra of G_3-$[Zn]_{24}$ **(9)** in DMSO-d^6 18

Figure S10. ^{1}H NMR spectrum of the first-generation Cu(II) metallodendrimer **(10)** recorded in CD_3OD……………………………………………………………...…19

Figure S11. UV–Vis spectra showing the d-d transition bands of Cu(II) metallodendrimers **10–12** 19

Figure S12. Degradation study of G_2-$[Zn]_{12}$ **(8)** by ^{1}H NMR in deuterated PBS (D_2O) containing 1% DMSO-d^6 at various time points, performed at pH 5.4 and 6.4 20

References..20

Synthesis Protocols

The bis-MPA-based dendrimers functionalized with cysteamine G_n-$[Cys]_m$) and used as starting materials were synthesized according to previously reported protocols.[1]

Synthesis of G_1-$[NH_2]_6$ **(1).** Triethylamine (TEA) (120.4 mg, 1.19 mmol) was added to a solution of dendrimer G_1-$[Cys]_6$ (250.0 mg, 0.18 mmol) in a 1:1 mixture of H_2O and dichloromethane (DCM). The reaction mixture was stirred for 2 h at room temperature. After reaction completion, the product was extracted 2 times with DCM, and the combined organic phases were dried over $MgSO_4$. Finally, the solvent was removed under vacuum to afford dendrimer **1** as yellow oil (194.9 mg, 91.3 %). $C_{51}H_{106}N_6O_{12}S_6$ (1185.8 g/mol). **^{1}H-NMR (CD_3OD)** δ 0.96 (t, $^3J_{(H-H)}$ = 7.4 Hz, 3H $\boldsymbol{H_3}CCH_2C(CH_2O\text{-})_3^{TMP}$); 1.21 (s, 9H, TMP-$O(C{=}O)C(C\boldsymbol{H_3})(CH_2O\text{-})_2$); 1.55 (m, 2H, $H_3CC\boldsymbol{H_2}C(CH_2O\text{-})_3^{TMP}$); 1.82 (m, 12H, $\text{-}OCH_2C\boldsymbol{H_2}CH_2S\text{-}$); 2.59 (t, $^3J_{(H-H)}$ = 7.2 Hz, 12H, $\text{-}OCH_2CH_2C\boldsymbol{H_2}S\text{-}$); 2.64 (t, $^3J_{(H-H)}$ = 6.6 Hz, 12H, $\text{-}SC\boldsymbol{H_2}CH_2NH_2$); 2.82 (t, $^3J_{(H-H)}$ = 6.6 Hz, 12H, $\text{-}SCH_2C\boldsymbol{H_2}NH_2$); 3.52 (m, overlapping signals, 24H, $\text{-}O(C{=}O)C(CH_3)(C\boldsymbol{H_2}O(CH_2)_3S\text{-})_2$ and $\text{-}OC\boldsymbol{H_2}CH_2CH_2S\text{-}$); 4.08 (s, 6H, $H_3CCH_2C(C\boldsymbol{H_2}O\text{-})_3^{TMP}$). **^{13}C-NMR ($CD_3OD$)** δ 8.1 ($H_3\boldsymbol{C}CH_2C(CH_2O\text{-})_3^{TMP}$); 18.7 (TMP-$O(C{=}O)C(\boldsymbol{C}H_3)(CH_2O\text{-})_2$); 24.3 ($H_3C\boldsymbol{C}H_2C(CH_2O\text{-})_3^{TMP}$); 29.2 ($\text{-}OCH_2CH_2\boldsymbol{C}H_2S\text{-}$); 31.0 ($\text{-}OCH_2\boldsymbol{C}H_2CH_2S\text{-}$); 35.4 ($\text{-}S\boldsymbol{C}H_2CH_2NH_2$); 41.3 ($\text{-}SCH_2\boldsymbol{C}H_2NH_2$); 43.2 ($H_3CCH_2\boldsymbol{C}(CH_2O\text{-})_3^{TMP}$); 50.1 (TMP-$O(C{=}O)\boldsymbol{C}(CH_3)(CH_2O\text{-})_2$); 64.6 ($H_3CCH_2C(\boldsymbol{C}H_2O\text{-})_3^{TMP}$); 70.1 ($\text{-}O\boldsymbol{C}H_2CH_2CH_2S\text{-}$); 74.3 (TMP-$O(C{=}O)C(CH_3)(\boldsymbol{C}H_2O)_2\text{-}$); 175.9 (TMP-$O(\boldsymbol{C}{=}O)C(CH_3)(CH_2O\text{-})_2$).

Synthesis of G_2-$[NH_2]_{12}$ (**2**). Second generation dendrimer **2** was synthesized following the same protocol as for dendrimer **1**, with the following reagents: G_2-$[Cys]_{12}$ (169.9 mg, 0.056 mmol) and TEA (74.8 mg, 0.74 mmol). Product **2** was obtained as a yellow oil (116.7 mg, 80.6%). $C_{111}H_{218}N_{12}O_{30}S_{12}$ (2585.7 g/mol). **^{1}H-NMR (CD_3OD)** δ 1.01 (t, $^3J_{(H-H)}$ = 7.3 Hz, 3H $\boldsymbol{H_3}CCH_2C(CH_2O\text{-})_3^{TMP}$); 1.19 (s, 18H, $\text{-}O(C{=}O)C(C\boldsymbol{H_3})(CH_2O(CH_2)_3S\text{-})_2$); 1.31 (s, 9H, TMP-$O(C{=}O)C(C\boldsymbol{H_3})(CH_2O\text{-})_2$); 1.60 (m, 2H, $H_3CC\boldsymbol{H_2}C(CH_2O\text{-})_3^{TMP}$); 1.83 (m, 24H, $\text{-}OCH_2C\boldsymbol{H_2}CH_2S\text{-}$); 2.59 (t, $^3J_{(H-H)}$ = 7.7 Hz, 24H, $\text{-}OCH_2CH_2C\boldsymbol{H_2}S\text{-}$); 2.64 (t, $^3J_{(H-H)}$ = 6.8 Hz, 24H, $\text{-}SC\boldsymbol{H_2}CH_2NH_2$); 2.81 (t, $^3J_{(H-H)}$ = 6.6 Hz, 24H, $\text{-}SCH_2C\boldsymbol{H_2}NH_2$); 3.52 (m, overlapping signals, 48H, $\text{-}O(C{=}O)C(CH_3)(C\boldsymbol{H_2}O(CH_2)_3S\text{-})_2$ and $\text{-}OC\boldsymbol{H_2}CH_2CH_2S\text{-}$); 4.17 (s, 6H, $H_3CCH_2C(C\boldsymbol{H_2}O\text{-})_3^{TMP}$); 4.30 (s, 12H, TMP-$O(C{=}O)C(CH_3)(C\boldsymbol{H_2}O\text{-})_2$). **^{13}C-NMR ($CD_3OD$)** δ 8.0 ($H_3\boldsymbol{C}CH_2C(CH_2O\text{-})_3^{TMP}$); 18.7 (TMP-$O(C{=}O)C(\boldsymbol{C}H_3)(CH_2O\text{-})_2$ and $\text{-}O(C{=}O)C(\boldsymbol{C}H_3)(CH_2O(CH_2)_3S\text{-})_2$); 24.5 ($H_3C\boldsymbol{C}H_2C(CH_2O\text{-})_3^{TMP}$); 29.4 ($\text{-}OCH_2CH_2\boldsymbol{C}H_2S\text{-}$); 31.1 ($\text{-}OCH_2\boldsymbol{C}H_2CH_2S\text{-}$); 36.1 (-

$S\boldsymbol{C}H_2CH_2NH_2$); 42.1 ($-SCH_2\boldsymbol{C}H_2NH_2$); 42.7 ($H_3CCH_2\boldsymbol{C}(CH_2O\text{-})_3^{TMP}$); 46.5 (TMP-$O(C{=}O)\boldsymbol{C}(CH_3)(CH_2O\text{-})_2$); 49.0 ($-O(C{=}O)\boldsymbol{C}(CH_3)(CH_2O(CH_2)_3S\text{-})_2$); 65.4 ($H_3CCH_2C(\boldsymbol{C}H_2O\text{-})_3^{TMP}$); 65.6 (TMP-$O(C{=}O)C(CH_3)(\boldsymbol{C}H_2O\text{-})_2$); 71.5 ($-O\boldsymbol{C}H_2CH_2CH_2S$-); 74.2 ($-O(C{=}O)C(CH_3)(\boldsymbol{C}H_2O(CH_2)_3S\text{-})_2$); 173.5 (TMP-$O(\boldsymbol{C}{=}O)C(CH_3)(CH_2O\text{-})_2$); 175.2 ($-O(\boldsymbol{C}{=}O)C(CH_3)(CH_2O(CH_2)_3S\text{-})_2$).

Synthesis of G_3-[NH_2]$_{24}$ **(3)**. Third generation dendrimer **3** was synthesized following the same protocol as for dendrimer **1**, with the following reagents: G_3-[Cys]$_{24}$ (154.6 mg, 0.025 mmol) and TEA (66.8 mg, 0.66 mmol). Product **3** was obtained as yellow oil (124.8 mg, 92.7%). $C_{231}H_{446}N_{24}O_{66}S_{24}$ (5385.7 g/mol). **^{1}H-NMR (CD_3OD)** δ 1.04 (t, $^3J_{(H\text{-}H)}$ = 7.4 Hz, 3H $\boldsymbol{H_3}CCH_2C(CH_2O\text{-})_3^{TMP}$); 1.20 (s, 36H, $-O(C{=}O)C(C\boldsymbol{H_3})(CH_2O(CH_2)_3S\text{-})_2$); 1.30 (s, 18H, $-O(C{=}O)C(C\boldsymbol{H_3})(CH_2O\text{-})_2$) 1.38 (s, 9H, TMP-$O(C{=}O)C(C\boldsymbol{H_3})(CH_2O\text{-})_2$); 1.65 (m, 2H, $H_3CC\boldsymbol{H_2}C(CH_2O\text{-})_3^{TMP}$); 1.84 (m, 48H, $-OCH_2C\boldsymbol{H_2}CH_2S$-); 2.60 (m, 48H, -$OCH_2CH_2C\boldsymbol{H_2}S$-); 2.64 (m, 48H, $-SC\boldsymbol{H_2}CH_2NH_2$); 2.81 (m, 48H, $-SCH_2C\boldsymbol{H_2}NH_2$); 3.53 (m, overlapping signals, 96H, $-O(C{=}O)C(CH_3)(C\boldsymbol{H_2}O(CH_2)_3S\text{-})_2$ and $-OC\boldsymbol{H_2}CH_2CH_2S$-); 4.21 (s, 6H, $H_3CCH_2C(C\boldsymbol{H_2}O\text{-})_3^{TMP}$); 4.28 (s, 24H, $-O(C{=}O)C(CH_3)(C\boldsymbol{H_2}O\text{-})_2$); 4.38 (s, 12H, TMP-$O(C{=}O)C(CH_3)(C\boldsymbol{H_2}O\text{-})_2$). **^{13}C-NMR ($CD_3OD$)** δ 7.1 ($H_3\boldsymbol{C}CH_2C(CH_2O\text{-})_3^{TMP}$); 18.6 (TMP-$O(C{=}O)C(\boldsymbol{C}H_3)(CH_2O\text{-})_2$, $-O(C{=}O)C(\boldsymbol{C}H_3)(CH_2O\text{-})_2$ and -$O(C{=}O)C(\boldsymbol{C}H_3)(CH_2O(CH_2)_3S\text{-})_2$); 22.0 ($H_3C\boldsymbol{C}H_2C(CH_2O\text{-})_3^{TMP}$); 29.4 (-$OCH_2CH_2\boldsymbol{C}H_2S$-); 31.0 ($-OCH_2\boldsymbol{C}H_2CH_2S$-); 35.6 ($-S\boldsymbol{C}H_2CH_2NH_2$); 42.1 (-$SCH_2\boldsymbol{C}H_2NH_2$); 43.1 ($H_3CCH_2\boldsymbol{C}(CH_2O\text{-})_3^{TMP}$); 48.5 (TMP-$O(C{=}O)\boldsymbol{C}(CH_3)(CH_2O\text{-})_2$, -$O(C{=}O)\boldsymbol{C}(CH_3)(CH_2O\text{-})_2$ and $-O(C{=}O)\boldsymbol{C}(CH_3)(CH_2O(CH_2)_3S\text{-})_2$); 65.7 ($H_3CCH_2C(\boldsymbol{C}H_2O\text{-})_3^{TMP}$, TMP-$O(C{=}O)C(CH_3)(\boldsymbol{C}H_2O\text{-})_2$ and $-O(C{=}O)C(CH_3)(\boldsymbol{C}H_2O\text{-})_2$); 71.2 ($-O\boldsymbol{C}H_2CH_2CH_2S$-); 73.7 ($-O(C{=}O)C(CH_3)(\boldsymbol{C}H_2O(CH_2)_3S\text{-})_2$); 171.8 (TMP-$O(\boldsymbol{C}{=}O)C(CH_3)(CH_2O\text{-})_2$); 173.7 ($-O(\boldsymbol{C}{=}O)C(CH_3)(CH_2O\text{-})_2$); 175.0 (-$O(\boldsymbol{C}{=}O)C(CH_3)(CH_2O(CH_2)_3S\text{-})_2$).

Synthesis of G_1-[NPh]$_6$ **(4)**. 2-pyridinecarboxaldehyde (64.3 mg, 0.60 mmol) was added to a solution of dendrimer **1** (124.2 mg, 0.10 mmol) in DCM. The reaction mixture was stirred at room temperature and in the presence of $MgSO_4$ for 15 minutes. Upon completion, $MgSO_4$ was filtered, and the solvent was evaporated under vacuum to afford compound **4** as a yellow-brown oil (163.3 mg, 94.9%). $C_{87}H_{122}N_{12}O_{12}S_6$ (1720.4 g/mol).**^{1}H-NMR (DMSO-d^6)** δ 0.78 (m, 3H $\boldsymbol{H_3}CCH_2C(CH_2O\text{-})_3^{TMP}$); 1.03 (s, 9H, TMP-$O(C{=}O)C(C\boldsymbol{H_3})(CH_2O\text{-})_2$); 1.36 (m, 2H, $H_3CC\boldsymbol{H_2}C(CH_2O\text{-})_3^{TMP}$); 1.67 (m, 12H, -$OCH_2C\boldsymbol{H_2}CH_2S$-); 2.50 (m, overlap with DMSO signal, 12H, $-OCH_2CH_2C\boldsymbol{H_2}S$-); 2.76 (t,

$^3J_{(H-H)}$ = 6.7 Hz, 12H, $-SC\boldsymbol{H_2}CH_2NCPh$); 3.39 (m, overlapping signals, 24H, $-O(C=O)C(CH_3)(C\boldsymbol{H_2}O-)_2$ and $-OC\boldsymbol{H_2}CH_2CH_2S-$); 3.76 (t, $^3J_{(H-H)}$ = 6.8 Hz, 12H, $-SCH_2C\boldsymbol{H_2}NCPh$); 3.91 (s, 6H, $H_3CCH_2C(C\boldsymbol{H_2}O-)_3^{TMP}$); 7.43 (m, 6H, $-NCHC\boldsymbol{H}CHCHC-^{Ar}$); 7.84 (m, 6H, $-NCHCHC\boldsymbol{H}CHC-^{Ar}$); 7.89 (m, 6H, $-NCHCHCHC\boldsymbol{H}C-^{Ar}$); 8.31 (s, 6H, $-N=C\boldsymbol{H}-$); 8.60 (m, 6H, $-NC\boldsymbol{H}CHCHCHC-^{Ar}$). **^{13}C-NMR (DMSO-d^6)** δ 7.66 ($H_3\boldsymbol{C}CH_2C(CH_2O-)_3^{TMP}$); 17.9 ($TMP-O(C=O)C(\boldsymbol{C}H_3)(CH_2O-)_2$); 22.7 ($H_3C\boldsymbol{C}H_2C(CH_2O-)_3^{TMP}$); 27.8 ($-OCH_2CH_2\boldsymbol{C}H_2S-$); 29.8 ($-OCH_2\boldsymbol{C}H_2CH_2S-$); 31.8 ($-S\boldsymbol{C}H_2CH_2NCPh$); 40.9 ($H_3CCH_2\boldsymbol{C}(CH_2O-)_3^{TMP}$); 48.4 ($TMP-O(C=O)\boldsymbol{C}(CH_3)(CH_2O-)_2$); 60.3 ($-SCH_2\boldsymbol{C}H_2NCPh$); 63.4 ($H_3CCH_2C(\boldsymbol{C}H_2O-)_3^{TMP}$); 69.5 ($-O\boldsymbol{C}H_2CH_2CH_2S-$); 72.2 ($TMP-O(C=O)C(CH_3)(\boldsymbol{C}H_2O-)_2$); 120.5 ($-NCHCHCH\boldsymbol{C}HC-^{Ar}$); 125.6 ($-NCH\boldsymbol{C}HCHCHC-^{Ar}$); 136.8 ($-NCHCH\boldsymbol{C}HCHC-^{Ar}$); 149.8 ($-N\boldsymbol{C}HCHCHCHC-^{Ar}$); 154.6 ($-NCHCHCHCH\boldsymbol{C}-^{Ar}$); 162.5 ($-N=\boldsymbol{C}H-$); 173.1 ($TMP-O(\boldsymbol{C}=O)C(CH_3)(CH_2O-)_2$).

Synthesis of G_2-[NPh]$_{12}$ **(5)**. Second generation dendrimer **5** was synthesized following the same protocol as for dendrimer **4**, with the following reagents: Dendrimer **2** (119.9 mg, 0.046 mmol) and 2-pyridinecarboxaldehyde (58.9 mg, 0.55 mmol). Product **5** was obtained as yellow-brown oil (156.2 mg, 92.9%). $C_{183}H_{254}N_{24}O_{30}S_{12}$ (3654.9 g/mol). **^{1}H-NMR (DMSO-d^6)** δ 0.82 (m, 3H, $\boldsymbol{H_3}CCH_2C(CH_2O-)_3^{TMP}$); 1.03 (s, 18H, $-O(C=O)C(C\boldsymbol{H_3})(CH_2O(CH_2)_3S-)_2$); 1.14 (s, 9H, $TMP-O(C=O)C(C\boldsymbol{H_3})(CH_2O-)_2$); 1.39 (m, 2H, $H_3CC\boldsymbol{H_2}C(CH_2O-)_3^{TMP}$); 1.62 (m, 24H, $-OCH_2C\boldsymbol{H_2}CH_2S-$); 2.50 (m, overlap with DMSO signal, 24H, $-OCH_2CH_2C\boldsymbol{H_2}S-$); 2.76 (t, $^3J_{(H-H)}$ = 6.68 Hz, 24H, $-SC\boldsymbol{H_2}CH_2NCPh$); 3.35 (m, overlapping signals, 48H, $-O(C=O)C(CH_3)(C\boldsymbol{H_2}O(CH_2)_3S-)_2$ and $-OC\boldsymbol{H_2}CH_2CH_2S-$); 3.77 (t, $^3J_{(H-H)}$ = 6.11 Hz, 24H, $-SCH_2C\boldsymbol{H_2}NCPh$); 4.04 (s, 6H, $H_3CCH_2C(C\boldsymbol{H_2}O-)_3^{TMP}$); 4.15 (s, 12H, $TMP-O(C=O)C(CH_3)(C\boldsymbol{H_2}O-)_2$); 7.49 (m, 12H, $-NCHC\boldsymbol{H}CHCHC-^{Ar}$); 7.82 (m, 12H, $-NCHCHC\boldsymbol{H}CHC-^{Ar}$); 7.91 (m, 12H, $-NCHCHCHC\boldsymbol{H}C-^{Ar}$); 8.32 (s, 12H, $-N=C\boldsymbol{H}-$); 8.60 (m, 12H, $-NC\boldsymbol{H}CHCHCHC-^{Ar}$). **^{13}C-NMR (DMSO-d^6)** δ 7.4 ($H_3\boldsymbol{C}CH_2C(CH_2O-)_3^{TMP}$); 17.0 ($TMP-O(C=O)C(\boldsymbol{C}H_3)(CH_2O-)_2$); 17.5 ($-O(C=O)C(\boldsymbol{C}H_3)(CH_2O(CH_2)_3S-)_2$); 22.2 ($H_3C\boldsymbol{C}H_2C(CH_2O-)_3^{TMP}$); 27.9 ($-OCH_2CH_2\boldsymbol{C}H_2S-$); 29.6 ($-OCH_2\boldsymbol{C}H_2CH_2S-$); 32.0 ($-S\boldsymbol{C}H_2CH_2NCPh-$); 41.0 ($H_3CCH_2\boldsymbol{C}(CH_2O-)_3^{TMP}$); 46.5 ($TMP-O(C=O)\boldsymbol{C}(CH_3)(CH_2O-)_2$); 48.0 ($-O(C=O)\boldsymbol{C}(CH_3)(CH_2O(CH_2)_3S-)_2$); 60.1 ($-SCH_2\boldsymbol{C}H_2NCPh-$); 63.61 ($H_3CCH_2C(\boldsymbol{C}H_2O-)_3^{TMP}$); 64.7 ($TMP-O(C=O)C(CH_3)(\boldsymbol{C}H_2O-)_2$); 69.3 ($-O\boldsymbol{C}H_2CH_2CH_2S-$); 71.3 ($-O(C=O)C(CH_3)(\boldsymbol{C}H_2O(CH_2)_3S-)_2$); 120.2 ($-NCHCHCH\boldsymbol{C}HC-^{Ar}$); 125.2 ($-NCH\boldsymbol{C}HCHCHC-^{Ar}$); 136.6 ($-NCHCH\boldsymbol{C}HCHC-^{Ar}$); 149.4 ($-N\boldsymbol{C}HCHCHCHC-^{Ar}$); 153.9 ($-NCHCHCHCH\boldsymbol{C}-^{Ar}$);

162.2 (-N=***C***H-); 171.4 (TMP-O(***C***=O)C(CH$_3$)(CH$_2$O-)$_2$); 173.1 (-O(***C***=O)C(CH$_3$)(CH$_2$O(CH$_2$)$_3$S-)$_2$).

Synthesis of G$_3$-[NPh]$_{24}$ **(6).** Third generation dendrimer **6** was synthesized following the same protocol as for dendrimer **4**, with the following reagents: Dendrimer **3** (84.7 mg, 0.016 mmol) and 2-pyridinecarboxaldehyde (40.7 mg, 0.38 mmol). Product **6** was obtained as a yellow-brown oil (107.5 mg, 89.4%). $C_{375}H_{518}N_{48}O_{66}S_{24}$ (7517.2 g/mol). **^{1}H-NMR (DMSO-d^6)** δ 0.82 (m, 3H ***H$_3$***CCH$_2$C(CH$_2$O-)$_3$^{TMP}); 1.01 (s, 36H, -O(C=O)C(C***H$_3$***)(CH$_2$O(CH$_2$)$_3$S-)$_2$); 1.12 (s, 18H, -O(C=O)C(C***H$_3$***)(CH$_2$O-)$_2$) 1.18 (s, 9H, TMP-O(C=O)C(C***H$_3$***)(CH$_2$O-)$_2$); 1.41 (m, 2H, H$_3$CC***H$_2$***C(CH$_2$O-)$_3$^{TMP}); 1.66 (m, 48H, -OCH$_2$C***H$_2$***CH$_2$S-); 2.47 (m, overlap with DMSO signal, 48H, -OCH$_2$CH$_2$C***H$_2$***S-); 2.75 (m, 48H, -SC***H$_2$***CH$_2$NCPh); 3.34 (m, overlapping signals, 96H, -O(C=O)C(CH$_3$)(C***H$_2$***O(CH$_2$)$_3$S-)$_2$ and -OC***H$_2$***CH$_2$CH$_2$S-); 3.75 (m, 48H, -SCH$_2$C***H$_2$***CPh); 3.96 (s, 6H, H$_3$CCH$_2$C(C***H$_2$***O-)$_3$^{TMP}); 4.12 (s, 24H, -O(C=O)C(CH$_3$)(C***H$_2$***O-)$_2$); 4.21 (s, 12H, TMP-O(C=O)C(CH$_3$)(C***H$_2$***O-)$_2$); 7.39 (m, 24H, -NCHC***H***CHCHC-^{Ar}); 7.79 (m, 24H, -NCHCHC***H***CHC-^{Ar}); 7.88 (m, 24H, -NCHCHCHC***H***C-^{Ar}); 8.29 (s, 24H, -N=C***H***-); 8.57 (m, 24H, -NC***H***CHCHCHC-^{Ar}). **^{13}C-NMR (DMSO-d^6)** δ 7.1 (H$_3$***C***CH$_2$C(CH$_2$O-)$_3$^{TMP}); 17.4 (TMP-O(C=O)C(***C***H$_3$)(CH$_2$O-)$_2$, -O(C=O)C(***C***H$_3$)(CH$_2$O-)$_2$ and -O(C=O)C(***C***H$_3$)(CH$_2$O(CH$_2$)$_3$S-)$_2$); 22.9 (H$_3$C***C***H$_2$C(CH$_2$O-)$_3$^{TMP}); 28.0 (-OCH$_2$CH$_2$***C***H$_2$S-); 29.2 (-OCH$_2$***C***H$_2$CH$_2$S-); 32.2 (-S***C***H$_2$CH$_2$NCPh); 43.1 (H$_3$CCH$_2$***C***(CH$_2$O-)$_3$^{TMP}); 46.5 (TMP-O(C=O)***C***(CH$_3$)(CH$_2$O-)$_2$ and -O(C=O)***C***(CH$_3$)(CH$_2$O-)$_2$); 48.4 (-O(C=O)***C***(CH$_3$)(CH$_2$O(CH$_2$)$_3$S-)$_2$); 59.9 (-SCH$_2$***C***H$_2$NCPh); 63.4 (H$_3$CCH$_2$C(***C***H$_2$O-)$_3$^{TMP}, TMP-O(C=O)C(CH$_3$)(***C***H$_2$O-)$_2$ and -O(C=O)C(CH$_3$)(***C***H$_2$O-)$_2$); 69.1 (-O***C***H$_2$CH$_2$CH$_2$S-); 72.0 (-O(C=O)C(CH$_3$)(***C***H$_2$O(CH$_2$)$_3$S-)$_2$); 121.2 (-NCHCHCH***C***HC-^{Ar}); 125.7 (-NCH***C***HCHCHC-^{Ar}); 137.2 (-NCHCH***C***HCHC-^{Ar}); 149.7 (-N***C***HCHCHCHC-^{Ar}); 154.1 (-NCHCHCHCH***C***-^{Ar}); 162.8 (-N=***C***H-); 170.9 (TMP-O(***C***=O)C(CH$_3$)(CH$_2$O-)$_2$); 171.8 (-O(***C***=O)C(CH$_3$)(CH$_2$O-)$_2$); 173.2 (-O(***C***=O)C(CH$_3$)(CH$_2$O(CH$_2$)$_3$S-)$_2$).

Synthesis of G$_1$-[Zn]$_6$ **(7).** Dendrimer **4** (75.4 mg, 43.8 μmol) was dissolved in MeOH, and a solution of $ZnCl_2$ (36.8 mg, 0.27 mmol) in the same solvent was added dropwise. The reaction mixture was stirred overnight at room temperature. Upon completion, a brown precipitated appeared and the supernatant was removed. The precipitate was washed 3 times with MeOH and then dried, to afford dendrimer **7** was as a brown solid (90.4 mg, 78.0%). $C_{87}H_{144}Cl_{12}N_{12}O_{18}S_6Zn_6$ (2646.1 g/mol). **^{1}H-NMR (DMSO-d^6)** δ 0.81

(t, $^{3}J_{(H-H)}$ = 7.0 Hz, 3H ***H₃***CCH₂C(CH₂O-)₃^{TMP}); 1.09 (s, 9H, TMP-O(C=O)C(C***H₃***)(CH₂O-)₂); 1.40 (m, 2H, H₃CC***H₂***C(CH₂O-)₃^{TMP}); 1.67 (m, 12H, -OCH₂C***H₂***CH₂S-); 2.47 (m, 12H, -OCH₂CH₂C***H₂***S-); 2.69 (m, 12H, -SC***H₂***CH₂NCPh); 3.39 (m, overlapping signals, 24H, -O(C=O)C(CH₃)(C***H₂***O-)₂ and -OC***H₂***CH₂CH₂S-); 3.75 (m, 12H, -SCH₂C***H₂***NCPh); 3.95 (s, 6H, H₃CCH₂C(C***H₂***O-)₃^{TMP}); 7.77 (m, 6H, -NCHC***H***CHCHC-^{Ar}); 8.03 (m, 6H, -NCHCHCHC***H***C-^{Ar}); 8.20 (m, 6H, -NCHCHC***H***CHC-^{Ar}); 8.61 (s, 6H, -N=C***H***-); 8.67 (m, 6H, -NC***H***CHCHCHC-^{Ar}). **^{13}C-NMR (DMSO-d^{6})** δ 7.7 (H₃***C***CH₂C(CH₂O-)₃^{TMP}); 17.5 (TMP-O(C=O)C(***C***H₃)(CH₂O-)₂); 22.6 (H₃C***C***H₂C(CH₂O-)₃^{TMP}); 27.5 (-OCH₂CH₂***C***H₂S-); 29.0 (-OCH₂***C***H₂CH₂S-); 30.8 (-S***C***H₂CH₂NCPh); 41.0 (H₃CCH₂***C***(CH₂O-)₃^{TMP}); 48.2 (TMP-O(C=O)***C***(CH₃)(CH₂O-)₂); 57.0 (-SCH₂***C***H₂NCPh); 63.0 (H₃CCH₂C(***C***H₂O-)₃^{TMP}); 69.2 (-O***C***H₂CH₂CH₂S-); 72.2 (TMP-O(C=O)C(CH₃)(***C***H₂O-)₂); 126.5 (-NCHCHCH***C***HC-^{Ar}); 128.0 (-NCH***C***HCHCHC-^{Ar}); 140.4 (-NCHCH***C***HCHC-^{Ar}); 149.2 (-N***C***HCHCHCHC-^{Ar}); 153.3 (-NCHCHCHCH***C***-^{Ar}); 162.2 (-N=***C***H-); 172.6 (TMP-O(***C***=O)C(CH₃)(CH₂O-)₂). **Elemental Analysis:** Cal.: %C, 39.49; %H, 5.10; %N, 6.35. Exp:. %C, 39.20; %H, 5.17; %N, 6.36.

Synthesis of G₂-[Zn]₁₂ **(8).** Second generation dendrimer **8** was synthesized following the same protocol as for dendrimer **7**, with the following reagents: **5** (54.6 mg, 15.4 µmol) and $ZnCl_2$ (25.9 mg, 0.19 mmol). Product **8** was obtained as a brown solid (84.8 mg, 73.6%). $C_{183}H_{278}Cl_{24}N_{24}O_{42}S_{12}Zn_{12}$ (5506.4 g/mol). **^{1}H-NMR (DMSO-d^{6})** δ 0.85 (m, 3H, ***H₃***CCH₂C(CH₂O-)₃^{TMP}); 1.07 (s, 18H, -O(C=O)C(C***H₃***)(CH₂O(CH₂)₃S-)₂); 1.17 (s, 9H, TMP-O(C=O)C(C***H₃***)(CH₂O-)₂); 1.45 (m, 2H, H₃CC***H₂***C(CH₂O-)₃^{TMP}); 1.67 (m, 24H, -OCH₂C***H₂***CH₂S-); 2.47 (m, overlap with DMSO signal, 24H, -OCH₂CH₂C***H₂***S-); 2.69 (m, 24H, -SC***H₂***CH₂NCPh); 3.38 (m, overlapping signals, 48H, -O(C=O)C(CH₃)(C***H₂***O(CH₂)₃S-)₂ and -OC***H₂***CH₂CH₂S-); 3.75 (m, 24H, -SCH₂C***H₂***NCPh); 4.05 (s, 6H, H₃CCH₂C(C***H₂***O-)₃^{TMP}); 4.16 (s, 12H, TMP-O(C=O)C(CH₃)(C***H₂***O-)₂); 7.77 (m, 12H, -NCHC***H***CHCHC-^{Ar}); 8.02 (m, 12H, -NCHCHCHC***H***C-^{Ar}); 8.18 (m, 12H, -NCHCHC***H***CHC-^{Ar}); 8.60 (s, 12H, -N=C***H***-); 8.67 (m, 12H, -NC***H***CHCHCHC-^{Ar}). **^{13}C-NMR (DMSO-d^{6})** δ 7.2 (H₃***C***CH₂C(CH₂O-)₃^{TMP}); 18.6 (TMP-O(C=O)C(***C***H₃)(CH₂O-)₂ and -O(C=O)C(***C***H₃)(CH₂O(CH₂)₃S-)₂); 22.0 (H₃C***C***H₂C(CH₂O-)₃^{TMP}); 28.1 (-OCH₂CH₂***C***H₂S-); 29.5 (-OCH₂***C***H₂CH₂S-); 31.3 (-S***C***H₂CH₂NCPh-); 41.3 (H₃CCH₂***C***(CH₂O-)₃^{TMP}); 46.7 (TMP-O(C=O)***C***(CH₃)(CH₂O-)₂); 48.2 (-O(C=O)***C***(CH₃)(CH₂O(CH₂)₃S-)₂); 57.1 (-SCH₂***C***H₂NCPh-); 63.7 (H₃CCH₂C(***C***H₂O-)₃^{TMP}); 64.8 (TMP-O(C=O)C(CH₃)(***C***H₂O-)₂); 69.5 (-O***C***H₂CH₂CH₂S-

); 74.4 (-O(C=O)C(CH$_3$)(***C***H$_2$O(CH$_2$)$_3$S-)$_2$); 126.6 (-NCHCHCH***C***HC-^{Ar}); 128.6 (-NCH***C***HCHCHC-^{Ar}); 140.6 (-NCHCH***C***HCHC-^{Ar}); 149.9 (-N***C***HCHCHCHC-^{Ar}); 156.8 (-NCHCHCHCH***C***-^{Ar}); 162.4 (-N=***C***H-); 172.4 (TMP-O(***C***=O)C(CH$_3$)(CH$_2$O-)$_2$); 173.5 (-O(***C***=O)C(CH$_3$)(CH$_2$O(CH$_2$)$_3$S-)$_2$). **Elemental Analysis:** Cal.: %C, 39.92; %H, 5.09; %N, 6.10. Exp.: %C, 39.72; %H, 4.96; %N, 6.02.

Synthesis of G_3-[Zn]$_{24}$ **(9).** Third generation dendrimer **9** was synthesized following the same protocol as for dendrimer **7**, with the following reagents: **6** (37.4 mg, 5.0 µmol) and $ZnCl_2$ (16.4 mg, 0.12 mmol). Product **9** was obtained as a brown solid (39.7 mg, 70.8%). $C_{375}H_{566}Cl_{48}N_{48}O_{90}S_{24}Zn_{24}$ (11227.1 g/mol). **^{1}H-NMR (DMSO-d^6)** δ 0.84 (m, 3H ***H$_3$***CCH$_2$C(CH$_2$O-)$_3$^{TMP}); 1.04 (s, 36H, -O(C=O)C(C***H$_3$***)(CH$_2$O(CH$_2$)$_3$S-)$_2$); 1.12 (s, 18H, -O(C=O)C(C***H$_3$***)(CH$_2$O-)$_2$) 1.19 (s, 9H, TMP-O(C=O)C(C***H$_3$***)(CH$_2$O-)$_2$); 1.44 (m, 2H, H$_3$C***CH$_2$***C(CH$_2$O-)$_3$^{TMP}); 1.66 (m, 48H, -OCH$_2$C***H$_2$***CH$_2$S-); 2.46 (m, overlap with DMSO signal, 48H, -OCH$_2$CH$_2$C***H$_2$***S-); 2.70 (m, 48H, -SC***H$_2$***CH$_2$NCPh); 3.34 (m, overlapping of signals, 96H, -O(C=O)C(CH$_3$)(C***H$_2$***O(CH$_2$)$_3$S-)$_2$ and -OC***H$_2$***CH$_2$CH$_2$S-); 3.75 (m, 48H, -SCH$_2$C***H$_2$***CPh); 4.12 (m, overlapping signals, 42H, H$_3$CCH$_2$C(C***H$_2$***O-)$_3$^{TMP} ,-O(C=O)C(CH$_3$)(C***H$_2$***O-)$_2$ and TMP-O(C=O)C(CH$_3$)(C***H$_2$***O-)$_2$); 7.71 (m, 24H, -NCHC***H***CHCHC-^{Ar}); 7.99 (m, 24H, -NCHCHCHC***H***C-^{Ar}); 8.14 (m, 24H, -NCHCHC***H***CHC-^{Ar}); 8.56 (s, 24H, -N=C***H***-); 8.63 (m, 24H, -NC***H***CHCHCHC-^{Ar}). **^{13}C-NMR (DMSO-d^6)** δ 7.8 (H$_3$***C***CH$_2$C(CH$_2$O-)$_3$^{TMP}); 17.1 (TMP-O(C=O)C(***C***H$_3$)(CH$_2$O-)$_2$) 17.8 (-O(C=O)C(***C***H$_3$)(CH$_2$O-)$_2$) 18.6 (-O(C=O)C(***C***H$_3$)(CH$_2$O(CH$_2$)$_3$S-)$_2$); 22.3 (H$_3$C***C***H$_2$C(CH$_2$O-)$_3$^{TMP}); 27.7 (-OCH$_2$CH$_2$***C***H$_2$S-); 29.2 (-OCH$_2$***C***H$_2$CH$_2$S-); 31.0 (-S***C***H$_2$CH$_2$NCPh); 40.7 (H$_3$CCH$_2$***C***(CH$_2$O-)$_3$^{TMP}); 46.3 (TMP-O(C=O)***C***(CH$_3$)(CH$_2$O-)$_2$ and -O(C=O)***C***(CH$_3$)(CH$_2$O-)$_2$); 47.5 (-O(C=O)***C***(CH$_3$)(CH$_2$O(CH$_2$)$_3$S-)$_2$); 57.1 (-SCH$_2$***C***H$_2$NCPh); 63.5 (H$_3$CCH$_2$C(***C***H$_2$O-)$_3$^{TMP}, TMP-O(C=O)C(CH$_3$)(***C***H$_2$O-)$_2$ and -O(C=O)C(CH$_3$)(***C***H$_2$O-)$_2$); 69.0 (-O***C***H$_2$CH$_2$CH$_2$S-); 72.1 (-O(C=O)C(CH$_3$)(***C***H$_2$O(CH$_2$)$_3$S-)$_2$); 125.7 (-NCHCHCH***C***HC-^{Ar}); 128.2 (-NCH***C***HCHCHC-^{Ar}); 139.8 (-NCHCH***C***HCHC-^{Ar}); 149.2 (-N***C***HCHCHCHC-^{Ar}); 158.4 (-NCHCHCHCH***C***-^{Ar}); 162.2 (-N=***C***H-); 173.0 (TMP-O(***C***=O)C(CH$_3$)(CH$_2$O-)$_2$, -O(***C***=O)C(CH$_3$)(CH$_2$O-)$_2$ and -O(***C***=O)C(CH$_3$)(CH$_2$O(CH$_2$)$_3$S-)$_2$). **Elemental Analysis:** Cal.: %C, 40.12; %H, 5.08; %N, 5.99. Exp:. %C, 41.79; %H, 4.19; %N, 6.32.

Synthesis of G_1-[Cu]$_6$ **(10).** Dendrimer **4** (82.6 mg, 48.0 µmol) was dissolved in acetone, and a solution of $Cu(NO_3)_2 \cdot 3H_2O$ (70.1 mg, 0.29 mmol) in the same solvent was added dropwise, resulting in the formation of a dark-green precipitate. The reaction mixture was

stirred for 2 h at room temperature. The supernatant was then removed, and the precipitate was washed three times with acetone and dried, affording dendrimer **10** as a dark-green solid (87.5 mg, 61.7%). $C_{87}H_{134}Cu_6N_{24}O_{54}S_6$ (2953.8 g/mol). **Elemental Analysis:** Cal.: %C, 35.38; %H, 4.57; %N, 11.38. Exp:. %C, 32.46; %H, 4.43; %N, 11.38. **ICP-OES:** Cal.: %Cu, 12.91. **UV-VIS:** λ = 648 nm.

Synthesis of G_2*-[Cu]*$_{12}$ **(11)**. Second generation dendrimer **11** was synthesized following the same protocol as for **10**, with the following reagents: Dendrimer **5** (63.9 mg, 17.5 µmol) and $Cu(NO_3)_2{\cdot}3H_2O$ (50.7 mg, 0.21 mmol).Product **11** was obtained as a dark-green solid (69.1 mg, 64.5%). $C_{183}H_{278}Cu_{12}N_{48}O_{114}S_{12}$ (6121.7 g/mol). **Elemental Analysis:** Cal.: %C, 35.91; %H, 4.58; %N, 10.98. Exp:. %C, 34.48; %H, 4.36; %N, 10.31. **UV-VIS:** λ = 647 nm.

Synthesis of G_3*-[Cu]*$_{24}$ **(12).** Third generation dendrimer **12** was synthesized following the same protocol as for **10**, with the following reagents: **6** (41.3 mg, 5.49 µmol) and $Cu(NO_3)_2{\cdot}3H_2O$ (31.4 mg, 0.13 mmol). Product **12** was obtained as a dark-green solid (38.6 mg, 56.4%). $C_{375}H_{566}Cu_{24}N_{96}O_{234}S_{24}$ (12457.6 g/mol). **Elemental Analysis:** Cal.: %C, 36.16; %H, 4.58; %N, 10.79. %C, 35.98; %H, 3.79; %N, 10.90. **UV-VIS:** λ = 700 nm.

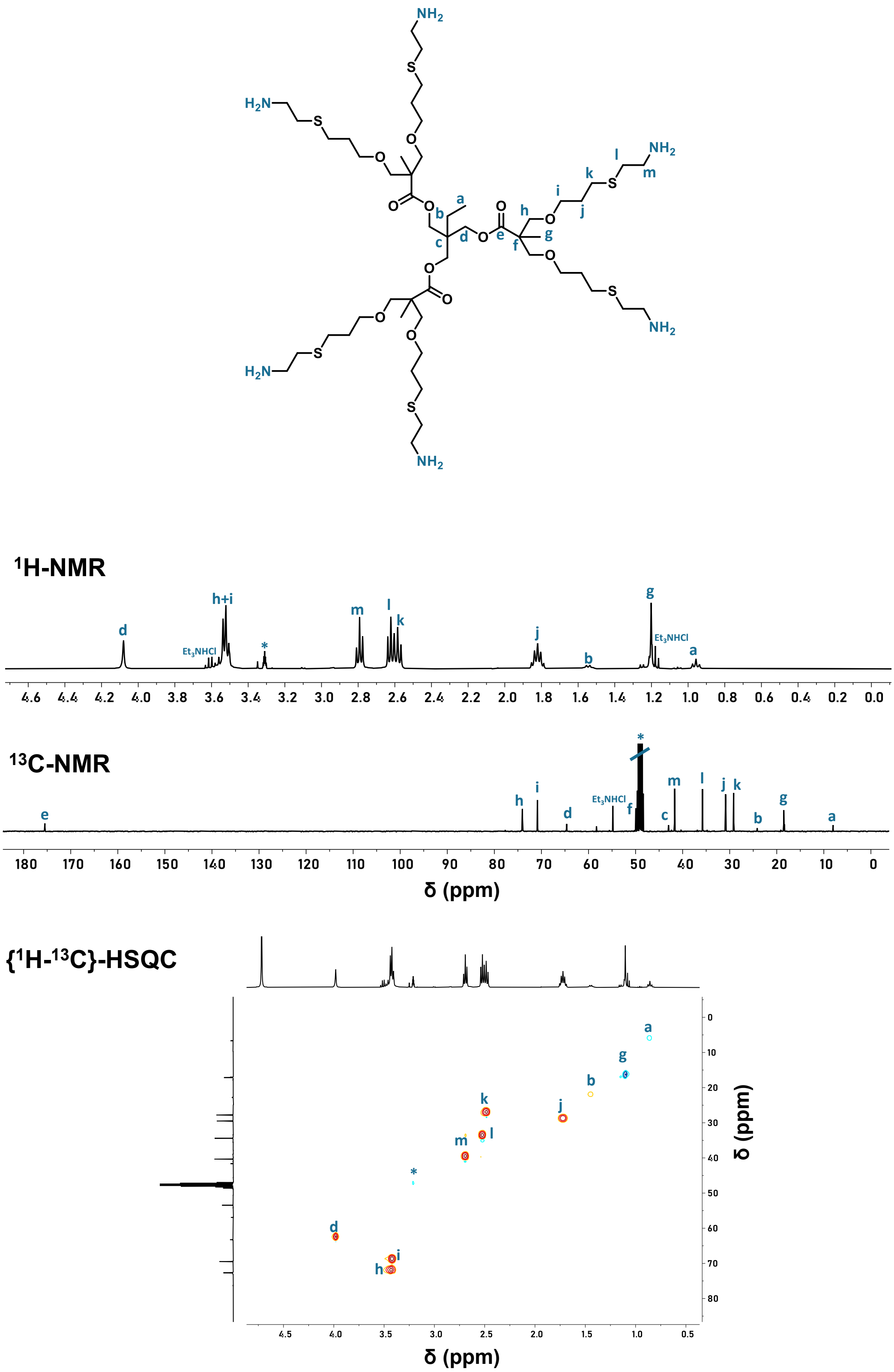

Figure S1. NMR spectra of G_1-$[NH_2]_6$ **(1)** in CD_3OD (*). Deuterated solvent signal in ^{13}C NMR has been truncated for clarity.

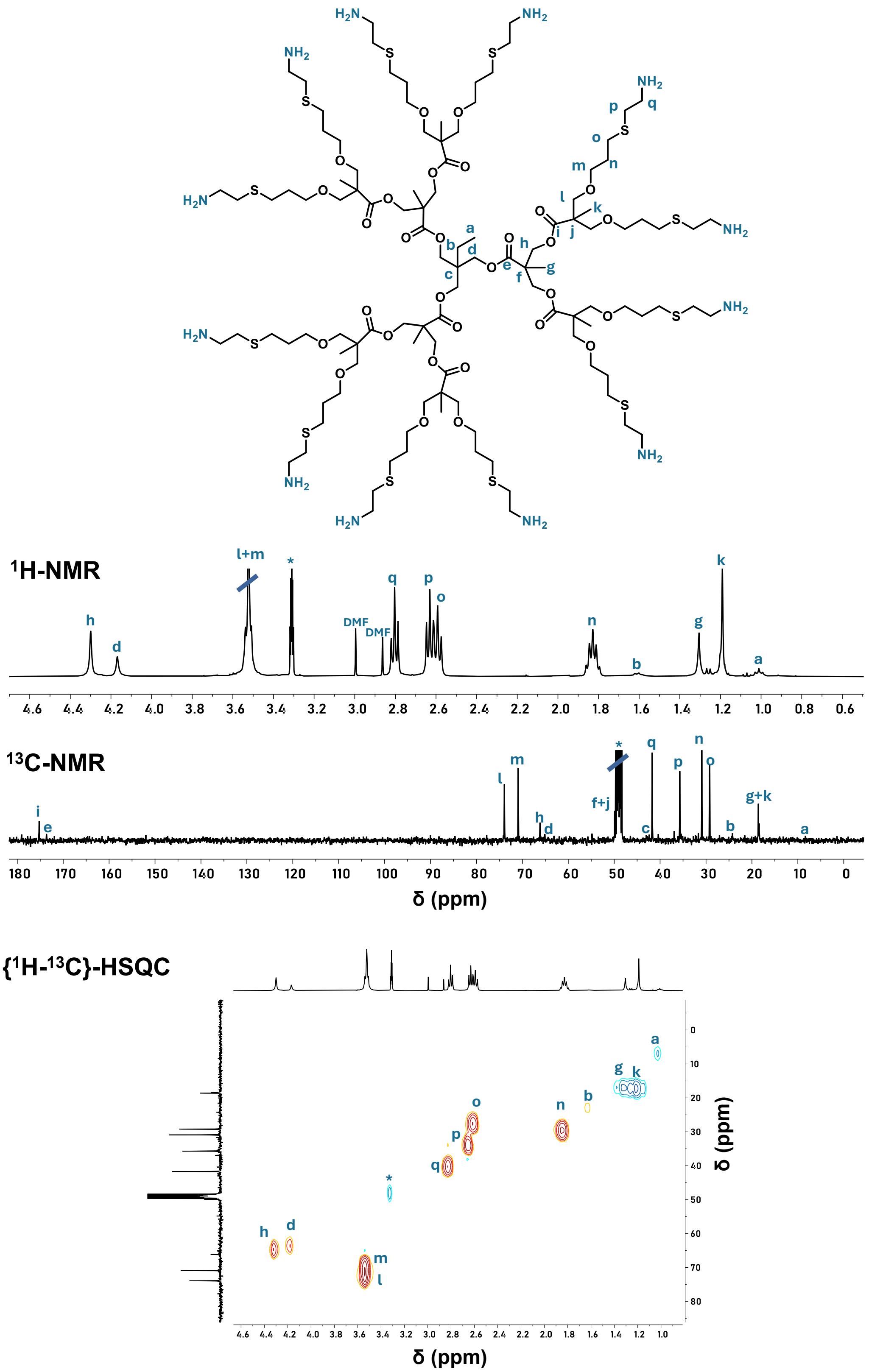

Figure S2. NMR spectra of G_2-$[NH_2]_{12}$ **(2)** in CD_3OD (*). Deuterated solvent signals in ^1H and ^{13}C NMR have been truncated for clarity.

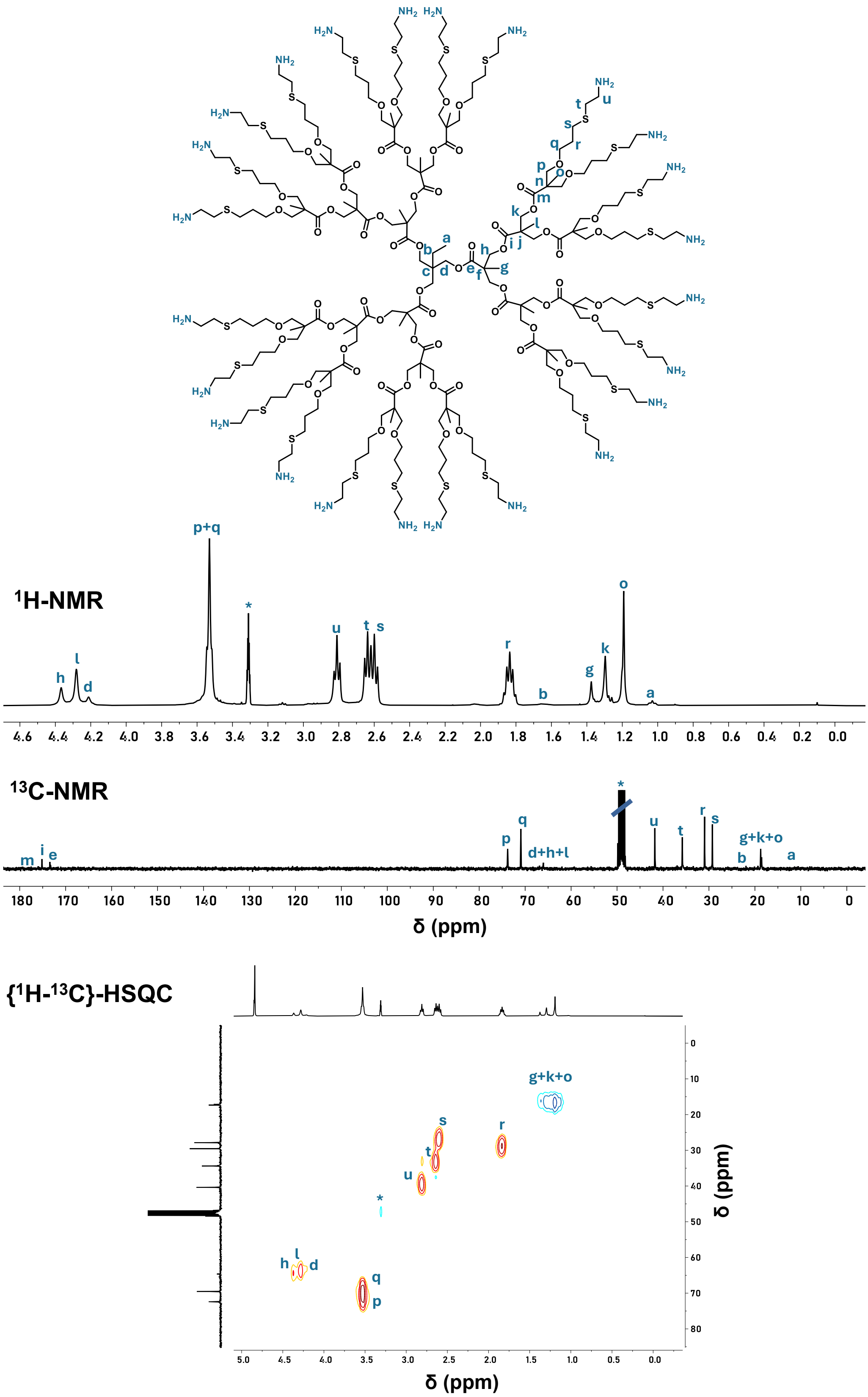

Figure S3. NMR spectra of G_3-$[NH_2]_{24}$ **(3)** in CD_3OD (*). Deuterated solvent signal in ^{13}C NMR has been truncated for clarity.

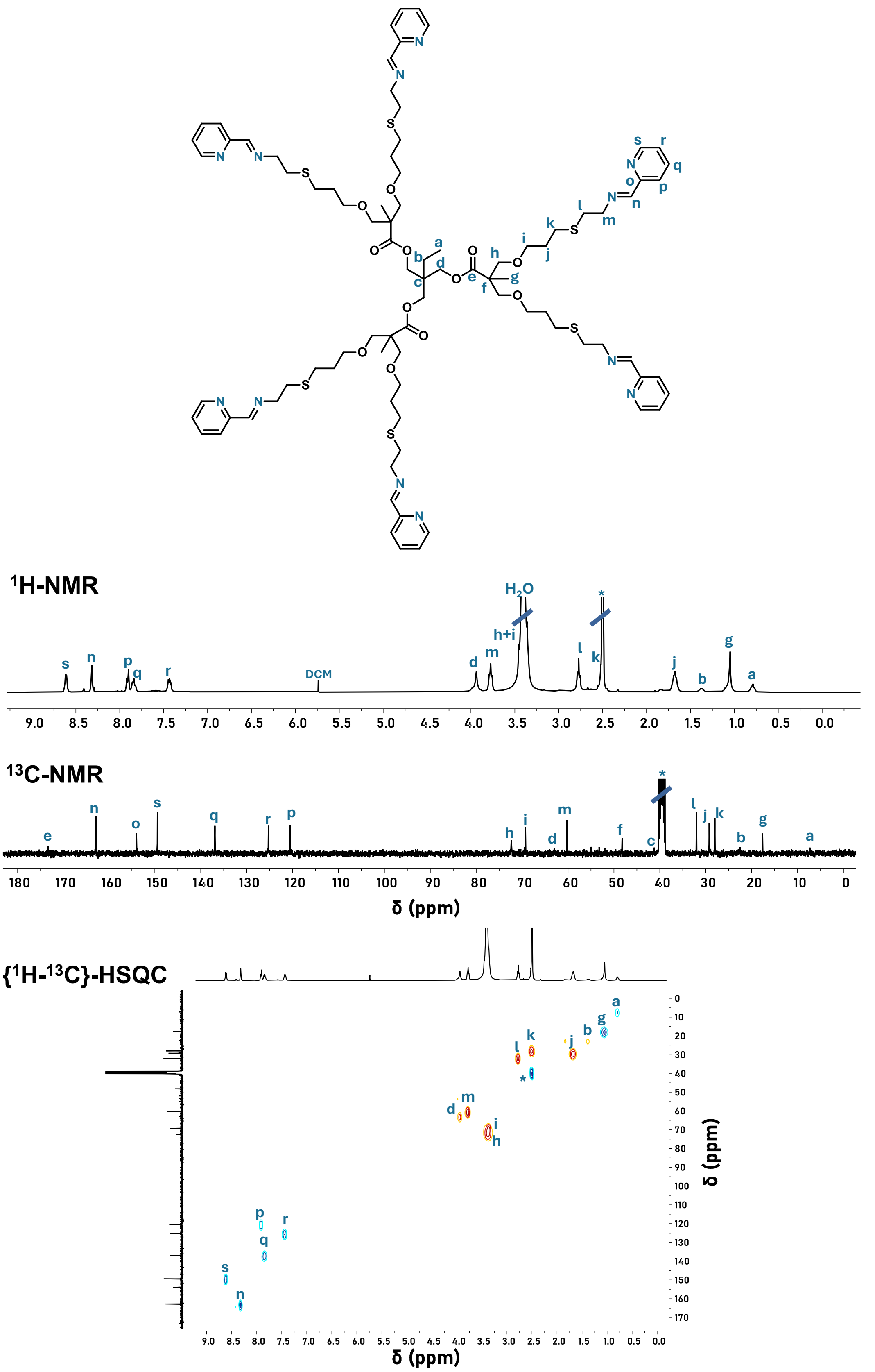

Figure S4. NMR spectra of G$_1$-[NPh]$_6$ **(4)** in DMSO-d^6 (*). Deuterated solvent signals in ^{1}H and ^{13}C NMR have been truncated for clarity.

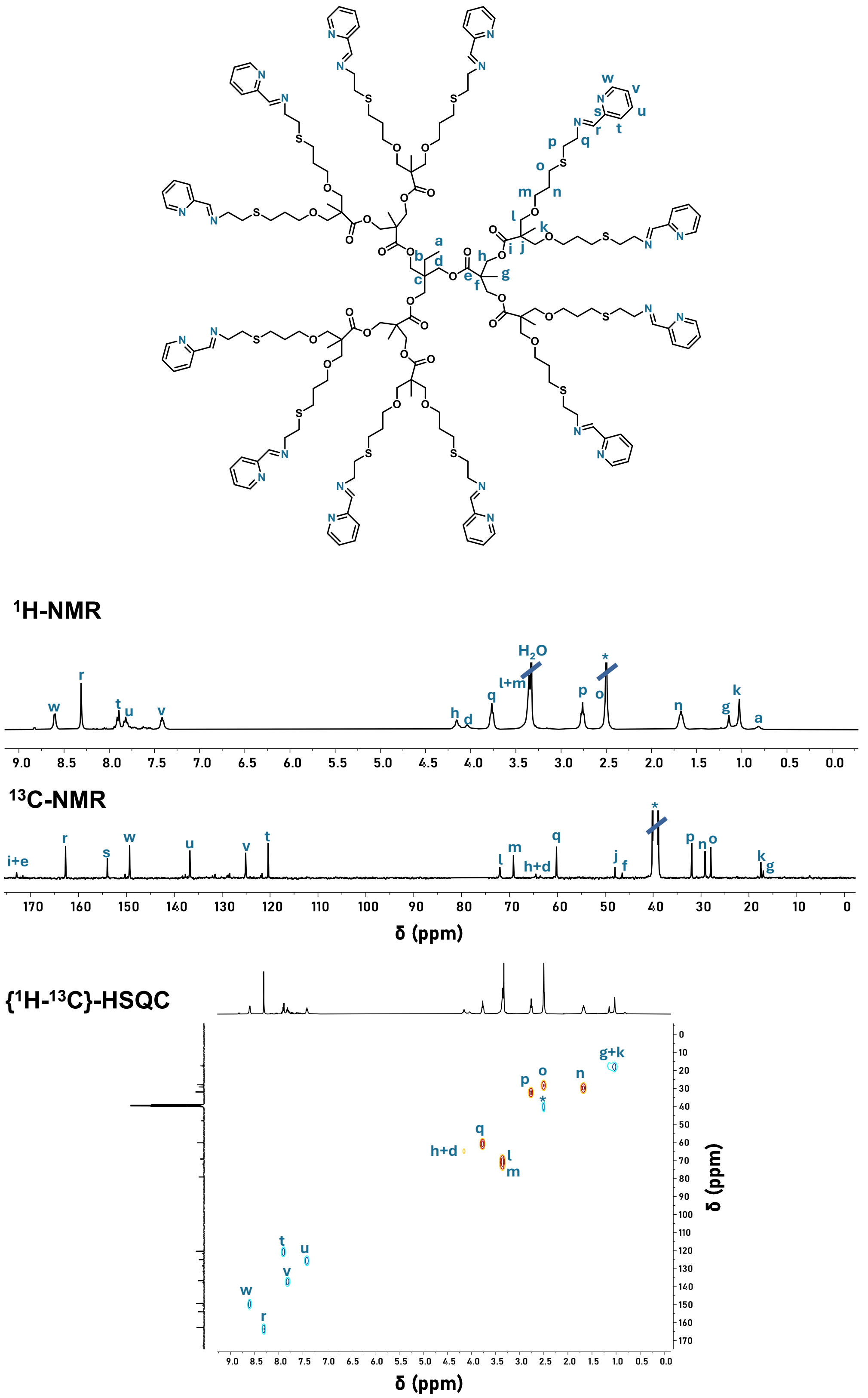

Figure S5. NMR spectra of G_2-$[NPh]_{12}$ **(5)** in DMSO-d^6 (*). Deuterated solvent signals in ^1H and ^{13}C NMR have been truncated for clarity.

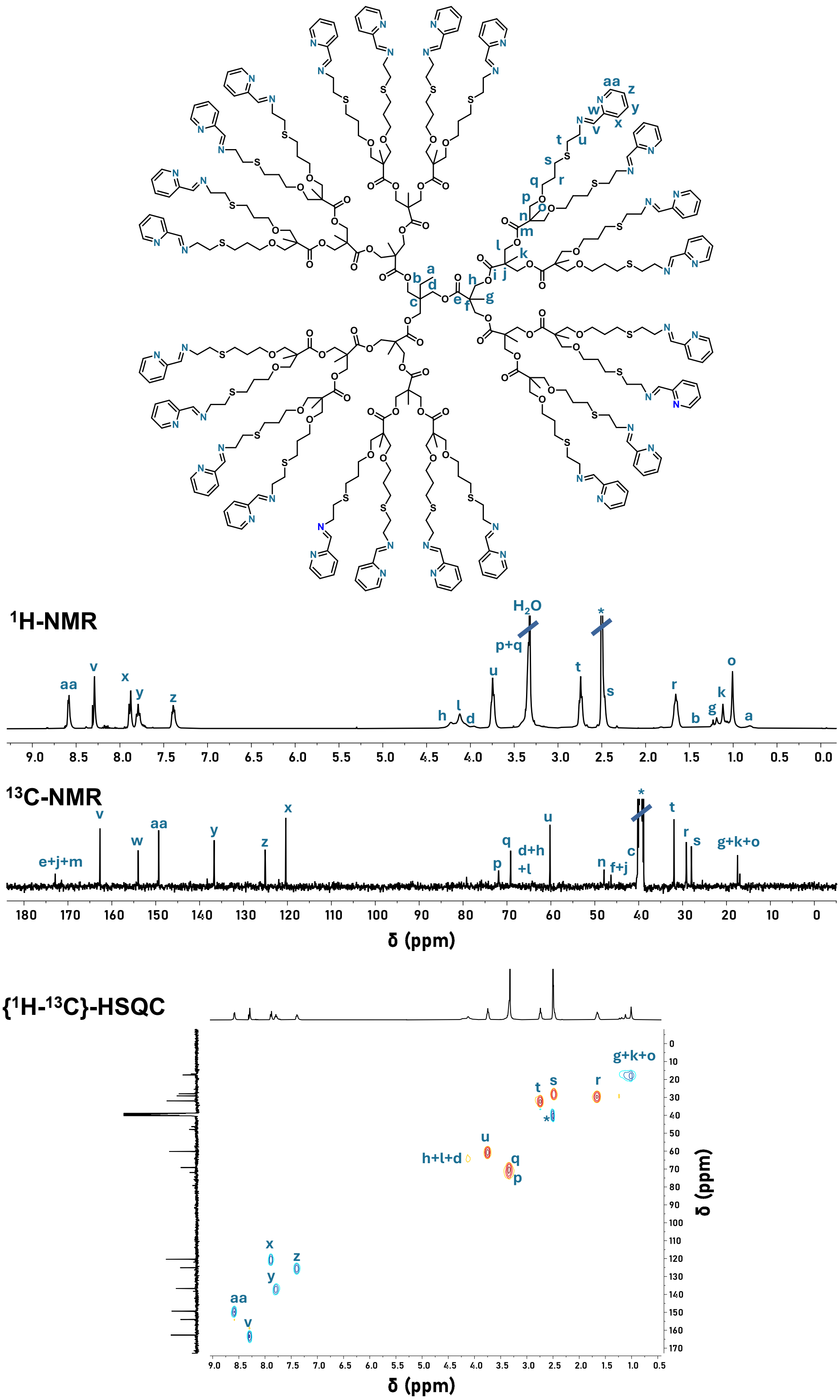

Figure S6. NMR spectra of G_3-$[NPh]_{24}$ **(6)** in DMSO-d^6 (*). Deuterated solvent signals in ^1H and ^{13}C NMR have been truncated for clarity.

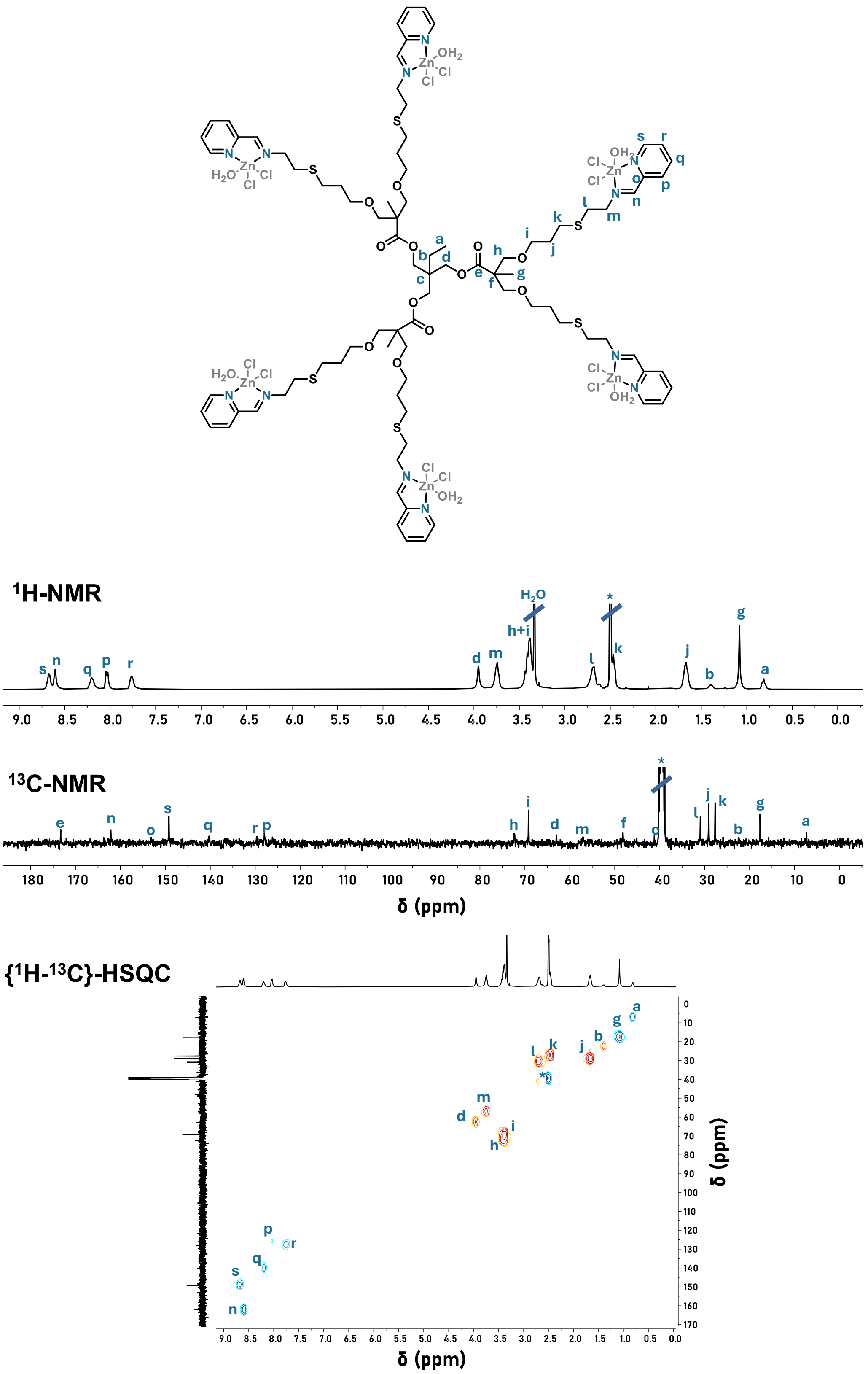

Figure S7. NMR spectra of G_1-$[Zn]_6$ **(7)** in DMSO-d^6 (*). Deuterated solvent signals in ^1H and ^{13}C NMR have been truncated for clarity.

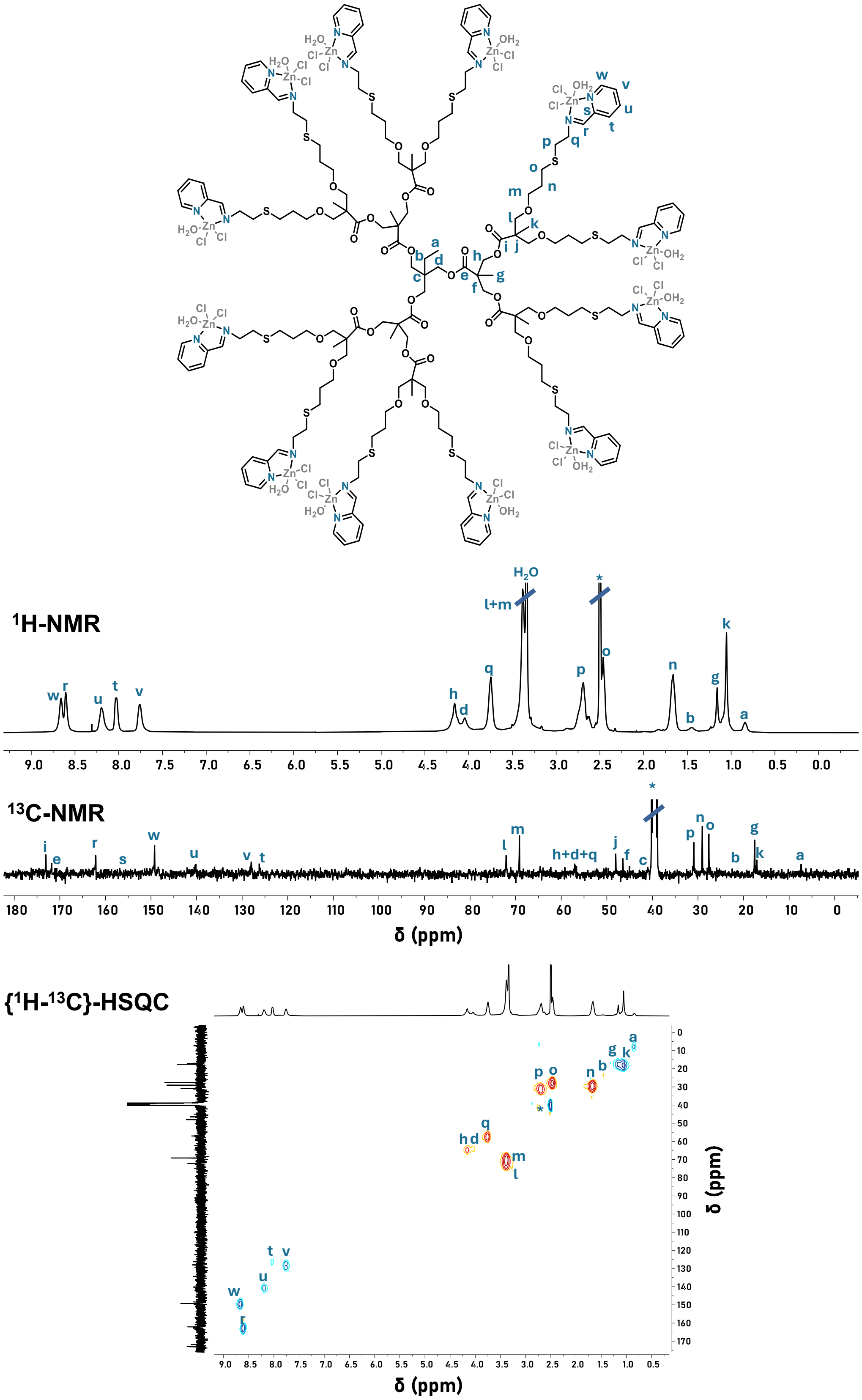

Figure S8. NMR spectra of G_2-$[Zn]_{12}$ **(8)** in DMSO-d^6 (*). Deuterated solvent signals in ^{1}H and ^{13}C NMR have been truncated for clarity.

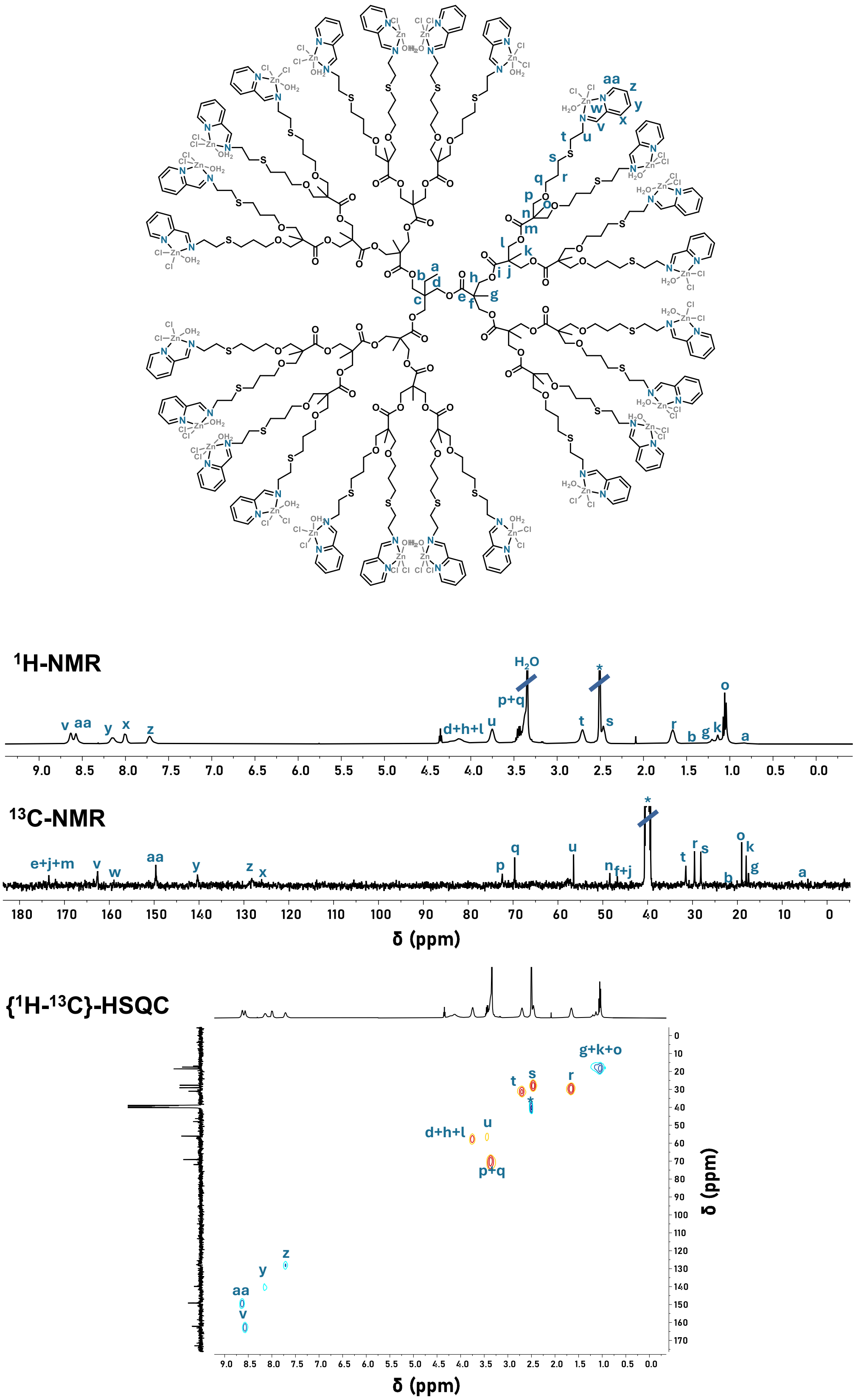

Figure S9. NMR spectra of G_3-$[Zn]_{24}$ **(9)** in DMSO-d^6 (*). Deuterated solvent signals in ^{1}H and ^{13}C NMR have been truncated for clarity.

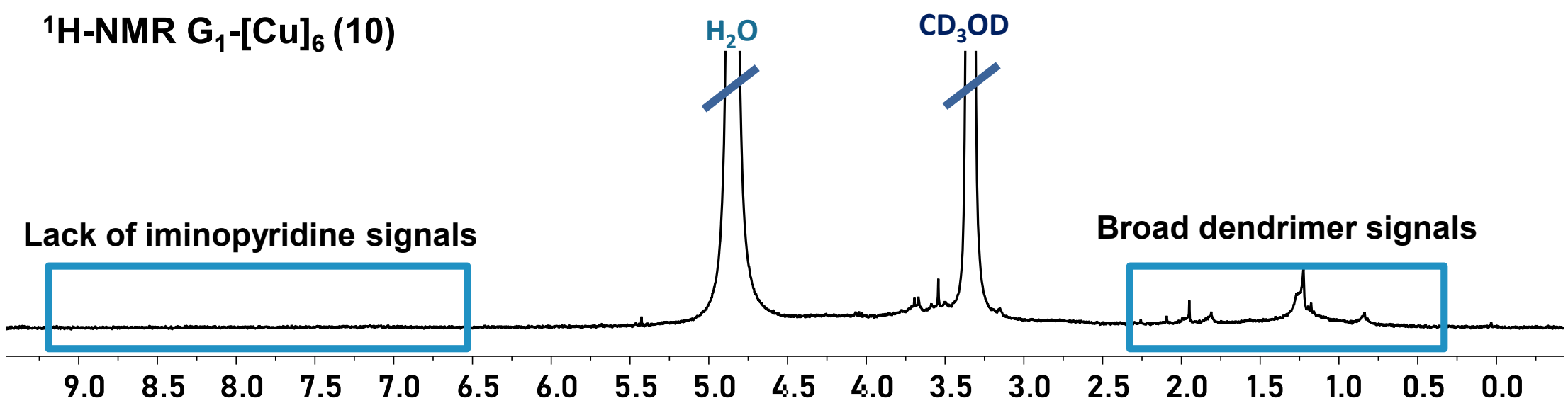

Figure S10. ^{1}H NMR spectrum of the first-generation Cu(II) metallodendrimer **(10)** recorded in CD_3OD.

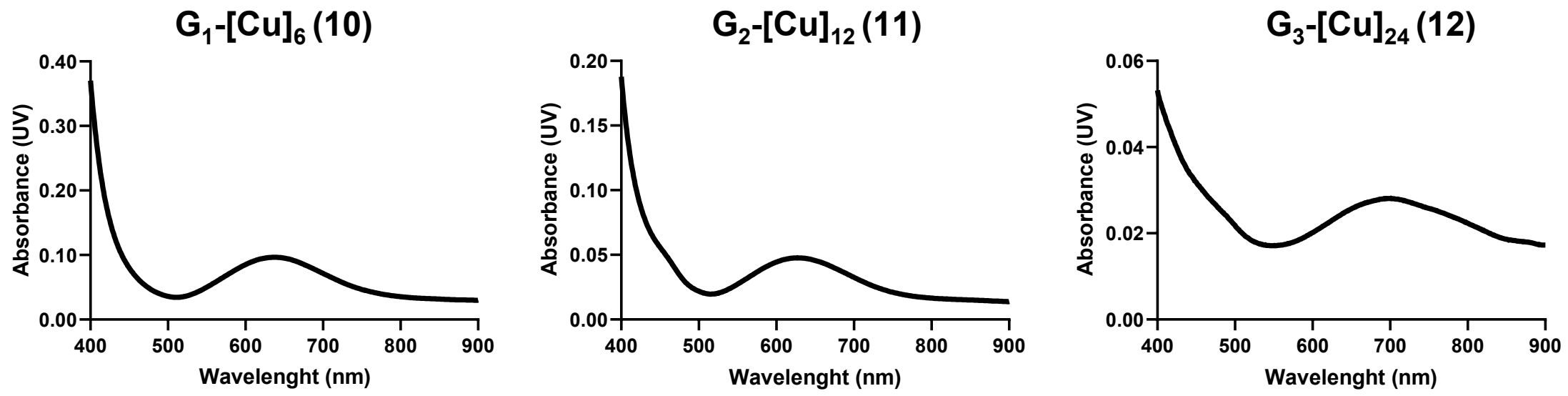

Figure S11. UV–Vis spectra showing the d-d transition bands of Cu(II) metallodendrimers **10–12**.

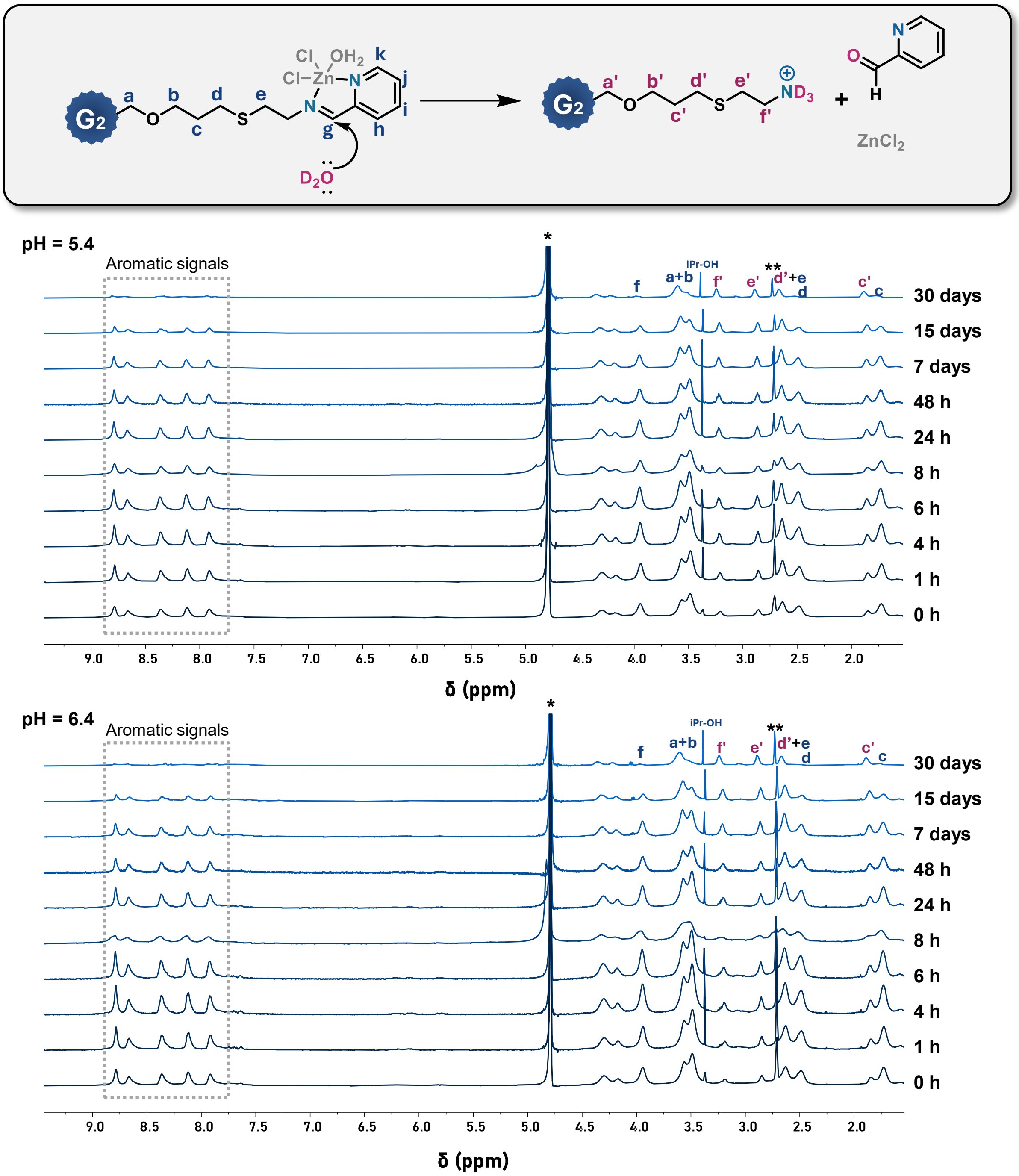

Figure S12. Degradation study of G_2-$[Zn]_{12}$ **(8)** by ^{1}H NMR in deuterated PBS (D_2O*) containing 1% DMSO-d_6 (**) at various time points, performed at pH 5.4 and 6.4.

References

1. Namata, F.; Sanz del Olmo, N.; Molina, N.; Malkoch, M. Synthesis and Characterization of Amino-Functional Polyester Dendrimers Based on Bis-MPA with Enhanced Hydrolytic Stability and Inherent Antibacterial Properties. *Biomacromolecules* **2023,** *24* (2), 858-867. DOI: 10.1021/acs.biomac.2c01286
